# *In Planta* Functional Analysis and Subcellular Localization of the Oomycete Pathogen *Plasmopara viticola* Candidate RXLR Effector Repertoire

**DOI:** 10.3389/fpls.2018.00286

**Published:** 2018-04-13

**Authors:** Yunxiao Liu, Xia Lan, Shiren Song, Ling Yin, Ian B. Dry, Junjie Qu, Jiang Xiang, Jiang Lu

**Affiliations:** ^1^College of Food Science and Nutritional Engineering, China Agricultural University, Beijing, China; ^2^Center for Viticulture and Enology, School of Agriculture and Biology, Shanghai Jiao Tong University, Shanghai, China; ^3^Guangxi Crop Genetic Improvement and Biotechnology Laboratory, Guangxi Academy of Agricultural Sciences, Nanning, China; ^4^CSIRO Agriculture & Food, Urrbrae, SA, Australia

**Keywords:** *Plasmopara viticola*, RXLR effectors, function, localization, chloroplast, mitochondria, dual-targeting

## Abstract

Downy mildew is one of the most destructive diseases of grapevine, causing tremendous economic loss in the grape and wine industry. The disease agent *Plasmopara viticola* is an obligate biotrophic oomycete, from which over 100 candidate RXLR effectors have been identified. In this study, 83 candidate RXLR effector genes (*PvRXLRs*) were cloned from the *P. viticola* isolate “JL-7-2” genome. The results of the yeast signal sequence trap assay indicated that most of the candidate effectors are secretory proteins. The biological activities and subcellular localizations of all the 83 effectors were analyzed via a heterologous *Agrobacterium*-mediated *Nicotiana benthamiana* expression system. Results showed that 52 effectors could completely suppress cell death triggered by elicitin, 10 effectors could partially suppress cell death, 11 effectors were unable to suppress cell death, and 10 effectors themselves triggered cell death. Live-cell imaging showed that the majority of the effectors (76 of 83) could be observed with informative fluorescence signals in plant cells, among which 34 effectors were found to be targeted to both the nucleus and cytosol, 29 effectors were specifically localized in the nucleus, and 9 effectors were targeted to plant membrane system. Interestingly, three effectors PvRXLR61, 86 and 161 were targeted to chloroplasts, and one effector PvRXLR54 was dually targeted to chloroplasts and mitochondria. However, western blot analysis suggested that only PvRXLR86 carried a cleavable *N*-terminal transit peptide and underwent processing *in planta*. Many effectors have previously been predicted to target organelles, however, to the best of our knowledge, this is the first study to provide experimental evidence of oomycete effectors targeted to chloroplasts and mitochondria.

## Introduction

Oomycetes are a group of straminipilous organisms, which are thought to have arisen from biflagellate, free-ranging predatory protists, and are most closely related to diatoms and brown algae, although they have evolved to possess characters similar to those of true fungi (Sekimoto et al., 2008; Beakes et al., 2012, 2014). Some oomycetes are highly destructive plant pathogens. For example, the potato late blight pathogen *Phytophthora infestans* not only caused the Irish famine in the nineteenth century but continues to affect modern agriculture (Dyer et al., 1993; Kamoun and Smart, 2005; Tyler et al., 2006; Fry, 2008; Yoshida et al., 2013).

The grapevine downy mildew pathogen *Plasmopara viticola* is an obligate oomycete belonging to the Peronosporaceae family, which causes severe damage to cultivated grapevines and wild species worldwide (Rouxel et al., 2013). As the most common varieties of wine and table grapes lack genetic resistance to this pathogen, control methods against *P. viticola* are mainly based on the frequent use of fungicides (Feechan et al., 2013). Due to the potential harmful impacts of agrochemicals on the environment and vineyard workers, finding alternative methods for the control of grapevine downy mildew is a high priority. However, the obligate biotrophic life cycle of *P. viticola* and the low efficiency of grapevine transformation make it difficult to investigate the molecular basis of the grapevine-downy mildew interaction (Perl et al., 1996; Kiefer et al., 2002; Kortekamp and Zyprian, 2003; Valsesia et al., 2005; Kortekamp, 2006; Dubresson et al., 2008; Kortekamp et al., 2008; Rossi and Caffi, 2012; Feechan et al., 2013; Rossi et al., 2013).

Most plant pathogens, including bacteria, fungi, oomycete, nematodes, and insects, are known to secrete a diverse array of low molecular weight proteins called effectors, which are thought to modulate plant immune responses and enhance pathogenicity (Jones and Dangl, 2006; Kamoun, 2006, 2007; Zipfel, 2008, 2014; Boller and He, 2009; Dodds and Rathjen, 2010; Wang et al., 2011). These effectors can be classified into two main categories, apoplastic, and cytoplasmic, based on their final locations in plant cells. The functions of most cytoplasmic effectors are poorly understood in comparison with those of apoplastic effectors, which often inhibit the activities of extracellular enzymes or interfere with the functions of host receptors (Damasceno et al., 2008; Ma et al., 2017). In contrast to most bacterial and fungal effectors, which rarely possess conserved motifs and domains, oomycete cytoplasmic effectors are modular proteins that carry *N*-terminal signal peptides followed by certain conserved motifs, notably the RXLR and LXLFLAK motifs. The RXLR motif defines a domain that is similar to a host-targeting translocation signal observed in the effectors secreted by malaria parasites, but the biological function of RXLR motif is still controversial (Birch et al., 2006; Dou et al., 2008a,b; Ellis and Dodds, 2011; Wawra et al., 2013, 2017). These so-called RXLR effectors have been identified in a diverse range of *Phytophthora* and downy mildew pathogens, but appear to be absent in necrotrophic pathogens, such as members of the *Pythium* genus and the animal pathogens belonging to the *Saprolegnia* genus (Lévesque et al., 2010; Krajaejun et al., 2011, 2014; Adhikari et al., 2013; Jiang et al., 2013). A number of RXLR effectors have been shown to suppress programmed cell death in plant cells (Bos et al., 2006; Dou et al., 2008a; Oh et al., 2009). However, the function of most RXLR effectors identified in obligate biotrophic pathogens, remains unclear.

Once inside host cells, effectors are transported to distinct compartments according to their sorting signals. Most effectors have been shown to be targeted to the nucleus, cytosol, plasma membrane, or endoplasmic reticulum (Caillaud et al., 2012; Lindeberg et al., 2012; Hicks and Galán, 2013). However, a few effectors have been reported to target chloroplasts and mitochondria. For example, four type-III bacterial effectors Hopl1, HopN1, HopK1, and AvrRps4 were reported to target chloroplasts (Jelenska et al., 2007; Rodríguez-Herva et al., 2012; Li et al., 2014). HopK1 and AvrRps4 are thought to target chloroplasts via their cleavable *N*-terminal transit peptides (Li et al., 2014), but similar *N*-terminal transit peptides appear to be lacking in Hopl1 and HopN1. The ToxA effector from the fungus *Pyrenophora tritici-repentis* not only targets the host chloroplasts, but also has the ability to cross the plant plasma membrane via the apoplastic space (Manning and Ciuffetti, 2005; Manning et al., 2007, 2009). More recently, the effectors CTP1, CTP2, and CTP3 secreted by the rust fungus *Melampsora larici-populina* were demonstrated to carry cleavable *N*-terminal transit peptides, which targeted them to chloroplasts (Petre et al., 2016). In addition, it was demonstrated that the type-III effector HopG1 was targeted to mitochondria and that it altered plant development, resulting in dwarfism, increased branching, and infertility (Block et al., 2010). However, oomycete effectors targeting chloroplasts and mitochondria have rarely been reported (Schornack et al., 2010; Caillaud et al., 2012; Stam et al., 2013).

*Plasmopara viticola* is thought to secrete a repertoire of effectors to modulate host physiological processes during infection, in a manner similar to that of other oomycete pathogens (Mestre et al., 2012, 2016; Yin et al., 2015; Xiang et al., 2016). Recently, the genome sequencing of the isolate “JL-7-2” revealed that *P. viticola* might potentially encode at least 100 candidate RXLR effectors (PvRXLRs) (Yin et al., 2017). The next challenge is to assess the functions of these effectors. However, as the pathogen is recalcitrant to stable transformation, it will be necessary to develop other approaches to determine the function of these *P. viticola* candidate effectors. The most widely used method for functional characterization of biotrophic pathogen effectors is based on a surrogate heterologous expression system, which facilitates the expression of effectors in plant cells directly via a bacterial type-three secretion system (TTSS) (Fabro et al., 2011), or agroinfiltration. Although the delivery of effectors into plant cells via the TTSS-mediated approach is efficient, the lack of post-translational modifications and the potential effect of co-expressed bacterial effectors may complicate the interpretation of results with this assay. In contrast, *Agrobacterium*-mediated heterologous expression in *Nicotiana benthamiana* allows for the transient expression of proteins in leaf cells, and a wide range of assays are available for conducting functional investigations. In this study, we successfully utilized the *Agrobacterium*-mediated heterologous expression strategy to investigate a total of 83 candidate PvRXLR effectors leading to a better understanding of the pathogenicity of this important pathogen.

## Materials and methods

### Microbial strains, plants, and culture conditions

*Escherichia coli* DH5α and *Agrobacterium tumefaciens* GV3101 were routinely grown in Luria-Bertani (LB) media containing the appropriate antibiotics at 37 and 30°C, respectively. DNA transformations of DH5α and GV3101 were conducted by using standard protocols for heat shock treatment and electroporation, respectively. The *P. viticola* isolate “JL-7-2” was maintained on detached leaves of *V. vinifera* cv. Thompson seedless at 18°C over a 16 h photoperiod. *Nicotiana benthamiana, V. vinifera* and *A. thaliana* plants were grown and maintained at 22°C in a controlled environment greenhouse with a 12 h photoperiod.

### Bioinformatics analysis

Classical nuclear localization signals, known conserved motifs and domains, and potential *N*-myristoylation sites were predicted using Motif Scan (http://myhits.isb-sib.ch/cgi-bin/motif_scan/). Signal peptide cleavage sites were predicted using the SignalP 4.1 server (http://www.cbs.dtu.dk/services/SignalP/; Petersen et al., 2011). Predictions of subcellular localization of effectors were made using WoLF PSORT (https://www.genscript.com/wolf-psort.html; Horton et al., 2007), ChloroP (http://www.cbs.dtu.dk/services/ChloroP/; Emanuelsson et al., 1999) and LOCALIZER (http://localizer.csiro.au/; Sperschneider et al., 2017). Protein parameters were calculated using ProtParam (http://web.expasy.org/protparam/; Walker, 2005). Protein sequence alignment was performed by using Protein BLAST (https://blast.ncbi.nlm.nih.gov/Blast.cgi).

### DNA and RNA manipulation

Grapevine leaves were surface-sterilized with chlorine bleach, followed by three washes in sterile water. The leaves were sprayed with a suspension containing 1 × 10^6^ sporangia/mL using an air brush, kept on 0.8% (w/v) agar plates, and incubated in a growth chamber at 18°C with a 16 h photoperiod. Leaves were sampled 3–4 days after infection and the total DNA and RNA extracted as described previously (Zhang et al., 2008). The cDNA was obtained using a one-step gDNA removal and cDNA synthesis supermix kit (TransGen Biotech, Beijing, China), according to the manufacturer's protocols.

### Gene cloning, RT-PCR, and vector construction

Full-length candidate effector genes (from ATG start codon to stop codon) were amplified from total genomic DNA by using 2 × High-Fidelity Master Mix (Tsingke Biotech, Beijing, China) (Table S2). In brief, 20 ng of total gDNA was amplified with an initial denaturation step at 98°C for 2 min; this was followed by 30 cycles at 98°C for 10 s, at 55°C for 15 s, and 72°C for 30 s. In the last cycle, a final elongation was carried out at 72°C for 1 min. For RT-PCR analysis, 50 ng of total RNA was used and amplified, as described above. PCR products were separated using 1.5% (w/v) TAE agarose gels and purified using a Gel Extraction Kit (Omega, Norcross, USA). The DNA fragments were inserted into a cloning vector pLB-Simple (Tiangen Biotech, Beijing, China) containing blunt-ends, and transformed into *E. coli* DH5α. Positive amplicons were confirmed by colony PCR. Five independent positive colonies carrying an insert of the expected size were selected for plasmid purification (Omega) and sequencing (Tsingke).

Sequences encoding mature PvRXLR proteins (i.e., without a signal peptide, in which translation is initiated from an artificial ATG codon) were introduced into linearized pGR106 (Potato virus X-based binary expression vector) (Chapman et al., 1992; Takken et al., 2000), pBI121, or pSuper1300 (Yang et al., 2010) using the In-Fusion cloning kit, according to the manufacturer's instructions (Tsingke). The gene sequence encoding the mature protein PvRXLR54 was introduced into a linearized pUC19-35S-GFP-RBS vector after *Xho* I and *BstB* I digestion, and the recombinant plasmids were purified by cesium chloride density gradient ultracentrifugation (Glisin et al., 1974). The DNA sequences of *ScCOX4*_1−29_ and *GmMan1*_1−49_ were synthesized by Tsingke Biological Technology and inserted into the pSuper1300 plasmid.

### Yeast signal sequence trap assay

The predicted DNA fragments encoding for the PvRXLR signal peptides were amplified by PCR, and purified using an Oligo DNA purification Kit (Shangon Biotech, Shanghai, China), and introduced into the linearized pSUC2 vector (pSUC2T7M13ORI) following *Eco*R I and *Xho* I (New England Biolabs, Ipswich, USA) digestion. The recombinant plasmids were transformed into the invertase-negative yeast strain YTK12 by using the lithium acetate method. Cells were plated onto CMD-W media (0.67% [w/v] yeast nitrogen base without amino acids, 0.075% [w/v] -Trp DO Supplement, 2% [w/v] sucrose, 0.1% [w/v] glucose, and 2% [w/v] agar) to select transformants; the positive transformants were re-confirmed by PCR and transferred to YPRAA media (1% [w/v] yeast extract, 2% [w/v] peptone, 2% [w/v] raffinose, and 2% [w/v] agar). The signal sequence trap assay was performed according to a method described by Oh et al. (2009)

### Functional verification of candidate PvRXLR effectors *in planta*

Four- to five-week-old *N. benthamiana* seedlings were used for agroinfiltration. *Agrobacterium tumefaciens* strain GV3101 carrying pGR106-PvRXLR effector plasmids or the negative control pGR106-GFP plasmid was cultured in LB liquid media (200 rpm, 30°C). The cells were collected by centrifugation (2,500 × g, 5 min), and resuspended with 10 mM MgCl_2_ until an OD_600_ of 0.4 was achieved, and then incubated at room temperature for 3 h before infiltration. For cell death suppression assays, *A. tumefaciens* strains carrying effectors and control GFP plasmids were first used to infiltrate the left and right sides of *N. benthamiana* leaves, respectively. One day later, the same areas of the leaves were re-infiltrated with *Agrobacterium* cultures carrying either the pGR106-BAX or pGR106-INF1 constructs. Co-expression of effectors and p19 was achieved by adjusting the OD_600_ of the two *Agrobacterium* cultures to 0.8, and mixing the culture solutions in a ratio of 1:1 (final OD_600_ of 0.4). The *Agrobacterium* culture carrying the pGR106-GFP vector was infiltrated at the same time as a control.

### Live-cell imaging

*Agrobacterium tumefaciens* cultures were infiltrated into *N. benthamiana* leaves in the manner described above with some modifications. For achieving subcellular localization of a single protein, the final OD_600_ was adjusted to 0.7; for co-localization studies, the final OD_600_ of two mixed *A. tumefaciens* cultures was adjusted to 0.5 (1.0 of each prior to mixing). Inoculated leaves were maintained under condition of low light and collected 36–72 h post-infiltration for analysis using a Leica TCS SP 8 confocal laser scanning platform (20 × air lens). The excitation wavelengths were set as follows: GFP and chlorophyll (488 nm) and mCherry (561 nm). The fluorescence signals for GFP, chlorophyll, and mCherry were detected at 505–525, 580–620 and 680–700 nm, respectively. Scanning was performed in a sequential mode when required, and all the images obtained were those of single optical sections, with cells exhibiting moderate fluorescence intensity levels. Sub-nuclear localizations were named according to the naming convention described by Caillaud et al. (2012).

### Protoplast transformation and production of transgenic plants

Protoplast transient expression assays were performed essentially according to the method described by Yoo et al. (2007) and Zhao et al. (2016), with some modifications. Grapevine leaves were cut into 0.5 mm strips and incubated in an enzyme solution (1.5% [w/v] Cellulase RS, 0.75% [w/v] Macerozyme R-10, 0.5 M mannitol, 10 mM MES at pH 5.7, 10 mM CaCl_2_ and 0.1% [w/v] BSA) for 5 h in the dark with gentle shaking (50 rpm, 25°C). Protoplasts were filtered through Miracloth and washed with W5 solution (154 mM NaCl, 125 mM CaCl_2_, 5 mM KCl, and 2 mM MES, pH 5.7). The pellets were collected by centrifugation at 100 × g for 5 min and resuspended in MMG solution (0.4 M mannitol, 15 mM MgCl_2_ and 4 mM MES, pH 5.7) at a concentration of 1 × 10^6^ cells/mL. The plasmid pUC19-35S-PvRXLR54-GFP (20 μg) was purified by ultracentrifugation and mixed with 200 μL protoplasts, followed by the addition of an equal volume of freshly prepared PEG solution (40% [w/v] PEG 3350, 0.4 M mannitol and 100 mM CaCl_2_). After incubation in PEG for 10 min at room temperature, the protoplasts were collected and washed with W5 and re-suspended in 1 mL WI solution (0.5 M mannitol, 20 mM KCl, 4 mM MES, pH 5.7), and incubated for 16 h at room temperature in the dark. Confocal microscopy was performed as described above.

The *A. tumefaciens* strain GV3101 carrying the pBI121-35S-PvRXLR54-GFP plasmid was used for stable transformation. Col-0 *A. thaliana* plants were transformed using the dipping method (Clough and Bent, 1998). The seeds were selected after cultivation in MS (without sucrose) medium containing 0.4% (w/v] phytagel and 50 μg/mL kanamycin. Transgenic plants were confirmed by GFP microscopy, transferred to soil and seeds were collected. T1 seedlings were analyzed by confocal microscopy. *Nicotiana benthamiana* (same strain as that used for agroinfiltration) was transformed using the leaf disc method (Horsch et al., 1985). The leaf discs were cultured on MS medium containing 0.8% (w/v) agar, 0.5 μg/mL IAA, 2 μg/mL NAA, 50 μg/mL kanamycin, and 200 μg/mL Timentin. Three weeks later, the shoots were transferred to MS medium containing 0.4% (w/v) phytagel, 0.2 μg/mL IAA, 10 μg/mL kanamycin, and 100 μg/mL Timentin. The regenerated seedlings were transferred to soil and cultivated for 1 week, after which confocal microscopy was performed. The reagents for plant tissue culture were purchased from Caisson Laboratories Company (Smithfield, UT, USA). The enzymes and other reagents used for achieving protoplast expression were purchased from Yakult (Minato-ku, Tokyo, Japan) and Sigma-Aldrich (St. Louis, MO, USA), respectively.

### Protein isolation and immunoblotting

Chloroplast protein extraction was carried out using the method described by Petre et al. (2016). Leaves were frozen in liquid nitrogen and ground into a powder. The lysis buffer (50 mM Tris-HCl pH 7.5, 150 mM KCl, 1 mM EDTA, 0.5% [v/v] Triton X-100, 1 mM DTT, 1 mM PMSF, 5 μM MG132, 1 × Protease Inhibitor Cocktail) was added to the mixture and it was kept on ice for 20 min. The extracts were centrifuged and supernatant was collected (14,000 × g, 20 min, 4°C). Protoplasts were collected (200 g, 2 min), mixed with 100 μL lysis buffer, and kept on ice for 5 min. Protoplast proteins were extracted as described above. The total proteins were loaded onto 12% SDS-PAGE gels and run for 1.5 h at 150 V. Gels were blotted onto a nitrocellulose membrane (PALL, Port Washington, NY, USA) for 1 h at 250 mA, and stained with Ponceau solution to confirm protein loading and transfer. Membranes were blocked in 5% [w/v] fat-free milk in 1 × TBST (0.5% [v/v] Tween-20), after which the primary monoclonal GFP antibody (TransGen) or polyclonal Rubisco large subunit (rbcL) antibody (Agrisera, Vännäs, Sweden) was added at a dilution of 1:10,000. The membrane was washed with TBST three times before the secondary anti-mouse or anti-rabbit IG-HRP raised in a goat (Sigma-Aldrich) was added at a 1:10,000 dilution. Blots were developed using the SuperSignal West Femto ECL kit (Thermo Scientific, Rockford, IL, USA).

## Results

### Cloning effector genes from the *P. viticola* isolate “JL-7-2”

**Figure 1:**
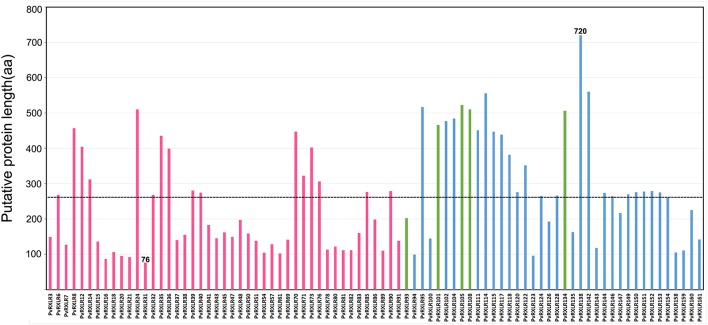
Analysis of amino acid length in 83 cloned candidate PvRXLR effectors. The dashed line indicates the average length of all effectors. Red bars indicate effectors whose expression had been previously detected by RNA-Seq analysis (Yin et al., 2015), blue bars indicate effectors whose expression is detected by RT-PCR analysis (in this study), and green bars indicate effectors whose expression during grapevine infection is unconfirmed.

A total of 83 candidate *PvRXLR* genes were successfully cloned from the *P. viticola* “JL-7-2” genome. Sequence analysis confirmed that 76 of these genes had the same length compared with the published genome sequence (Yin et al., 2017). In addition, three *PvRXLR* genes had insertions, which resulted in longer ORFs; and four *PvRXLR* genes had premature stop codons, which resulted in shorter ORFs. The presence of inconsistent ORFs was probably attributable to these effectors having highly similar paralogous genes, or a mismatched genome assembly. None of the cloned effector genes were found to contain introns. Of the 76 effectors with verified sequences, 49 encoded proteins which were identical to previously predicted sequences, while the remaining 27 exhibited SNPs, resulting in differences in one or several amino acids (Table S1). The predicted length of the PvRXLR proteins ranged from 76 to 720 aa, and the average length was 260 aa (Figure 1). Of the 83 cloned *PvRXLR* genes, 45 were previously shown to be expressed in infected grapevine leaves by RNA-Seq analysis (Yin et al., 2015). To determine whether the remaining 38 candidate *PvRXLR* genes were transcribed, RT-PCR analysis was carried out on the total RNA extracted from “JL-7-2” infected grapevine leaves. The results confirmed that 33/38 could be amplified from cDNA (Figure S1). Thus, in total, 78 out of the 83 predicted *PvRXLR* genes (~94%) in the *P. viticola* “JL-7-2” genome have been confirmed to be expressed during grapevine leaf infections.

### Functional validation of predicted signal peptides of PvRXLR proteins

To functionally validate signal peptide predictions at the *N*-terminus of PvRXLR effectors, we used a genetic assay based on the requirement that yeast cells need to secrete invertase to be able to grow on raffinose media (Klein et al., 1996; Jacobs et al., 1997; Lee et al., 2006; Oh et al., 2009). To perform this assay, signal sequences of 58 PvRXLR effectors with S-score values ranging from 0.812 to 0.995 were randomly selected and cloned into the yeast invertase vector pSUC2. The pSUC2 vector carrying the signal peptide of the RXLR effector Avr1b from *Phytophthora sojae* (Shan et al., 2004) was used as the positive control (CK +), and the untransformed YTK12 was used as negative control (CK−). The results indicate that 49 out of the 58 (~85%) predicted PvRXLR effector signal peptides supported invertase secretion, and allowed the YTK12 strain to grow on YPRAA medium (Figure S2). These results confirm that the majority of candidate PvRXLR effectors are likely to be secreted from the pathogen into the host plant cell.

### PvRXLR effectors suppress programmed cell death in *N. benthamiana*

To identify PvRXLR effectors possibly having a role in suppressing plant immunity, we first infiltrated *A. tumefaciens* cultures carrying the pGR106 expression vector containing the 83 different PvRXLR gene sequences (minus their predicted signal peptide sequence) into *N. benthamiana* leaves. After 24 h, these leaves were re-infiltrated with cultures carrying PCD-inducing BAX (a mammalian pro-apoptotic member of the Bcl-2 family, which induces apoptosis-like cell death in *N. benthamiana*; Lacomme and Santa Cruz, 1999) or INF1 (a 10-kD extracellular elicitin from *P. infestans*, which induces a hypersensitive response in *N. benthamiana*) (Kamoun et al., 1998). The cell death phenotype was scored after 4–5 days.

**Figure 2:**
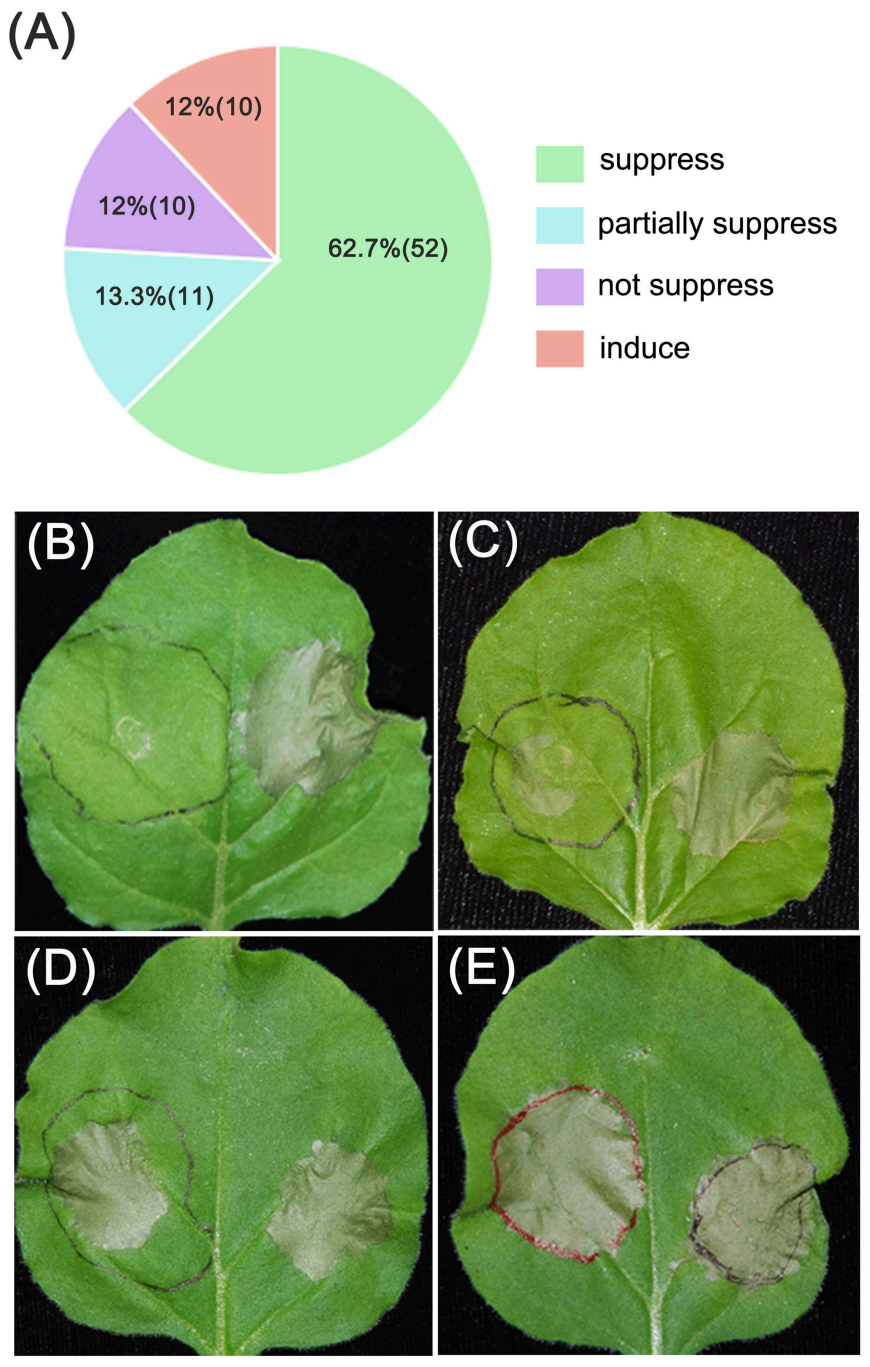
Summary of phenotypes observed upon expression of *PvRXLR* effectors in *N. benthamiana* leaves. Effector and GFP control constructs were agroinfiltrated into the left and right hand sides of *N. benthamiana* leaves, respectively. One day later, the original infiltration sites were re-infiltrated with INF1. Leaves were photographed and analyzed 4–6 days after infiltration. Similar results have been obtained from at least two independent experiments. **(A)** Summary of symptoms observed across all PvRXLR effectors tested. **(B–E)** Different symptom: suppressed (suppression of INF1-induced necrosis), partially suppressed (partial suppression of INF1-induced necrosis), not suppressed (no suppression of INF1-induced necrosis), and induced (an increase in induction of necrosis that was greater than that induced by INF1).

The results revealed that 52 PvRXLR effectors (~63%) could suppress INF1- and BAX-induced cell death completely (Figure 2B and Figure S3), while 10 effectors could only partially suppress cell death, as indicated by the weaker necrosis (Figure 2C and Figure S4A). However, 21 PvRXLR effectors were unable to suppress the necrosis triggered by the expression of INF1 and BAX (Figures 2D,E and Figures S4B,C). These results suggest that a majority of the PvRXLR effectors play a role in suppressing host immunity (Figure 2A and Figure S5).

### Certain PvRXLR effectors trigger programmed cell death in *N. benthamiana*

**Figure 3:**
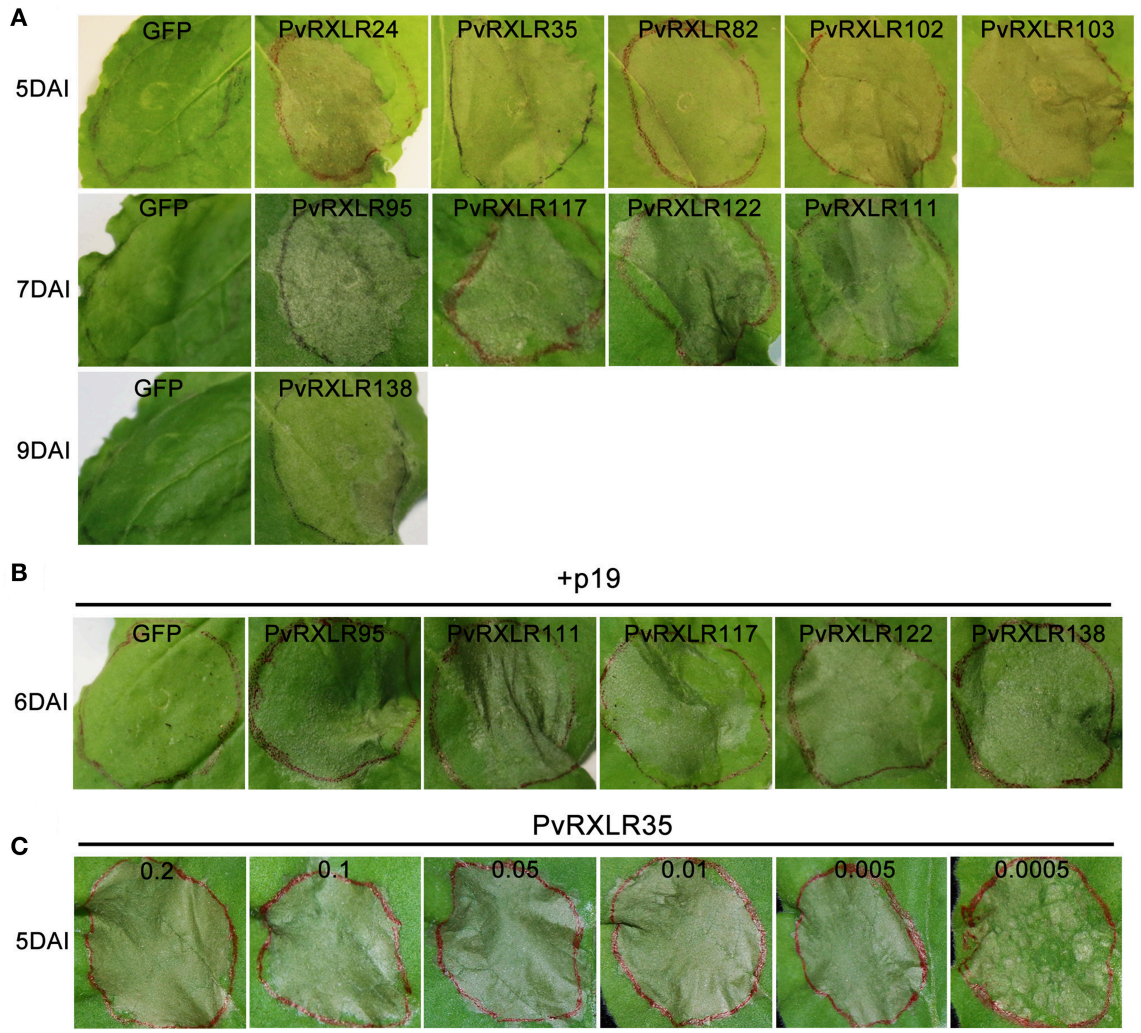
Specific PvRXLR effectors induce cell death in *N. benthamiana* leaves. **(A)** Individually expressed cell death-inducing effectors in *N. benthamiana* leaves at different days after infiltration are shown. **(B)** The five PvRXLR effectors showing the faster cell death-induction when co-infiltrated with p19. **(C)** Infiltration of a serial dilution (OD600 0.2–0.0005) of *Agrobacterium* containing PvRXLR35 expression construct into *N. benthamiana* leaves. Leaves have been photographed 5 days after inoculation.

The necrotic areas observed for 13 out of the 21 effectors that were unable to suppress INF1-triggered cell death were larger than those observed for the INF1 control (Figure 2E and Figure S4C). This result suggested that these PvRXLR effectors themselves trigger cell death. To test this hypothesis, *A. tumefaciens* strains carrying the pGR106 expression vector containing these 13 different PvRXLR effector gene sequences were agroinfiltrated into *N. benthamiana* leaves. The results showed that 10 out of the 13 non-suppressing effectors could trigger cell death independently, but that the speed of induction of necrosis varied markedly (Figure 3A). Five effectors induced clear necrosis on day 5, four at day 6, and one at day 9 after inoculation. It has previously been reported that the induction of cell death in *N. benthamiana*, by the *P. infestans* RXLR effector PexRD2,was dose-dependent (Oh et al., 2009). To determine if the rate of onset of necrosis induced by the different PvRXLR effectors was related to protein expression levels, the five weaker cell death effectors (PvRXLR95, 117, 122, 111, and 138) were co-expressed with p19, a suppressor of post-transcriptional gene silencing, which is known to increase gene expression in the agroinfiltration assay (Voinnet et al., 2003). The results showed that the onset of necrosis was accelerated for these PvRXLR effectors, when tested in the presence of p19 (Figure 3B), suggesting that effector protein levels were associated with the speed of initiation of programmed cell death. Interestingly, PvRXLR35 induced necrosis much more strongly than any of the other PvRXLR effectors, as indicated by the fact that strong necrosis could still be observed when very low concentrations of *Agrobacterium* cultures (i.e., OD600 of 0.0005) were used (Figure 3C). The amino acid sequences of these 10 PCD-inducing PVRXLR effectors were analyzed using Protein BLAST, but no conserved domains or homologous proteins were found, suggesting that these effectors were specific to *P. viticola*.

### Subcellular localization of PvRXLR effectors *in planta*

**Figure 4:**
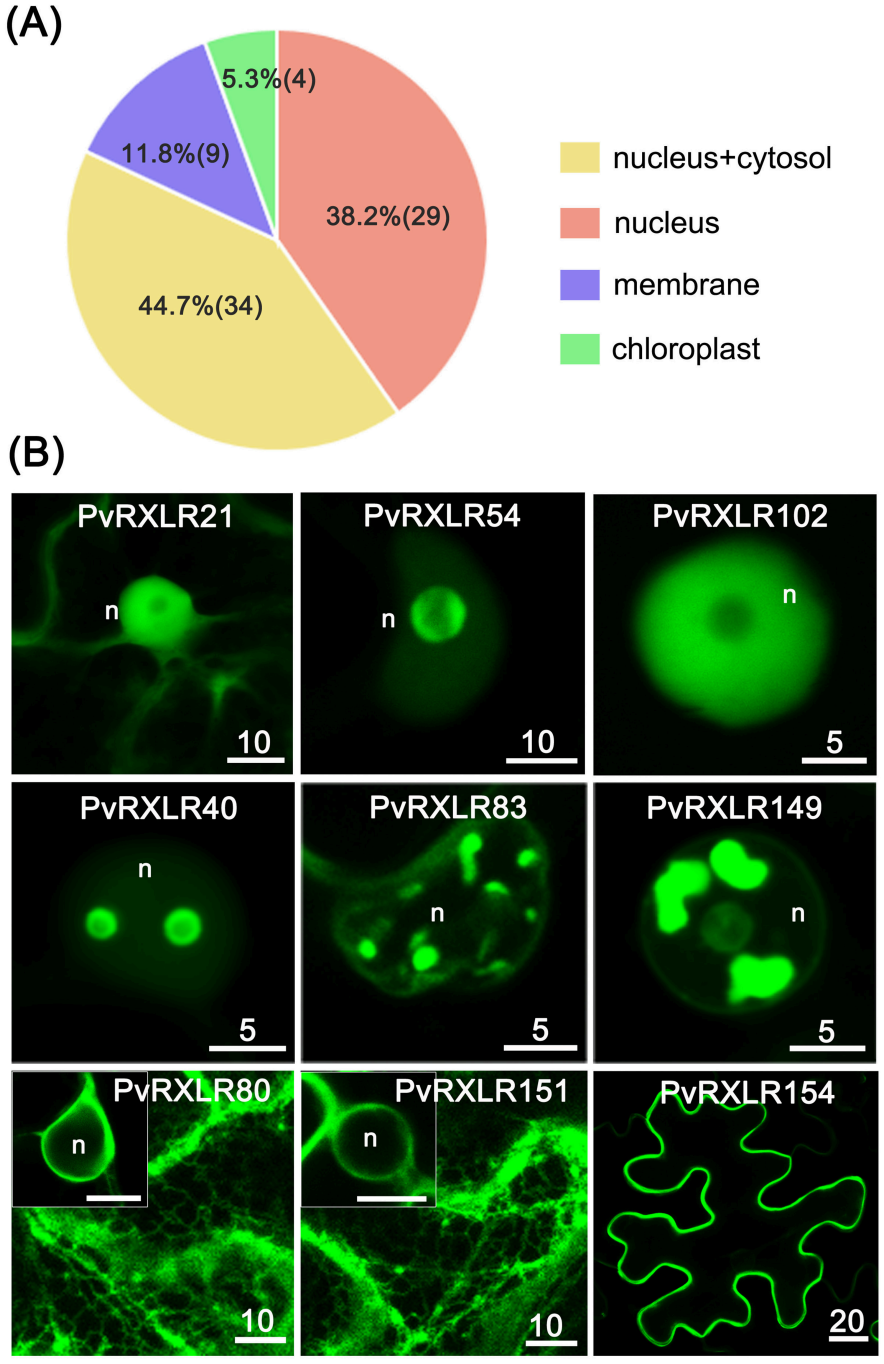
Subcellular localization of PvRXLR effectors in *N. benthamiana* leaf cells. Effector-GFP fusion constructs are agroinfiltrated into *N. benthamiana* leaves, and the accumulation and distribution pattern of fluorescent protein-tagged effectors are analyzed by confocal microscopy 36–72 h post-infiltration. **(A)** A summary of statistics of subcellular localizations of candidate effectors. **(B)** Representative images of different subcellular localization patterns of GFP-tagged PvRXLR effectors: nuclear and cytosolic (PvRXLR21), nucleus specific (PvRXLR45 and 102), sub-nuclear (PvRXLR40, 83, and 149), endoplasmic reticulum specific (ER) (PvRXLR80 and 151), and plasma membrane (PvRXLR154) specific localization. Scale bar = 5–20 μm.

To characterize the subcellular localizations of these candidate PvRXLR effectors, C-terminal GFP-tagged constructs were generated and expressed in *N. benthamiana* leaves via agroinfiltration. Live-cell imaging indicated that 76 out of the 83 PvRXLR effectors examined generated informative fluorescence signals in plant cells (Figure 4A and Figure S6). Of these, 29 (~38%) PvRXLR effectors were targeted specifically to the nucleus (Figure 4B and Figure S7), and 34 (~45%) were distributed in both the nucleus and the cytosol (Figure 4B and Figure S8). *In silico* analysis (Motif Scan) indicated that only 24 of the analyzed effectors had canonical nuclear-localization signals (NLS). However, most of the PvRXLR effectors contained NLS-like stretches of amino acids enriched in positively charged residues (arginine and lysine), which were predicted to target the proteins to the nucleus (Table S1) by the LOCALIZER prediction program.

Interestingly, we noted that most PvRXLR effectors targeted to the nucleus showed complex patterns of sub-nuclear distribution. Specifically, 14 effectors were targeted to the nucleolus, of which three (PvRXLR45, 91, and 138) were accumulated at the margin of the nucleolus, but were absent within it (Figure 4B and Figure S9A). Eleven effectors accumulated in irregular structures within the nucleus; of these, one (PvRXLR40) localized to bubble-like structures, one (PvRXLR128) localized to fiber-like structures, four (PvRXLR24, 83, 111, and 115) localized to speckle-like structures and four (PvRXLR76, 81, 134, and 149) localized to bulk/chunk-like structures (Figure 4B and Figure S9B).

In addition to the nuclear-targeted effectors, nine PvRXLR effectors were targeted to the plant membrane system. Of these, PvRXLR80 and 151 were associated solely with the endoplasmic reticulum (ER), five (PvRXLR85, 90, 143, 153, and 154) were associated solely with the plasma membrane, and three (PvRXLR47 and 126) were found to be associated with other membranes (Figures 4B and Figure S10). Amino acid sequence analysis of these 9 membrane-associated PvRXLR effectors revealed that most of them were predicted to be potential targets of post-translational *N*-myristoylation modification, which facilitates protein-lipid interactions and plays an essential role in membrane targeting (Sessa et al., 1993; Johnson et al., 1994).

In order to validate the observed subcellular distribution of PvRXLR effectors in *N. benthamiana* transient assays, 15 effectors were randomly selected to generate stable Arabidopsis transgenic lines. All of the PvRXLR effectors showed the same subcellular localization in stably transformed Arabidopsis transgenic lines, as that observed in transient expression assays (Figure S11), demonstrating that protein localization data derived from *N. benthamiana* agroinfiltration were highly reliable.

### Selected PvRXLR effectors are targeted to chloroplasts

**Figure 5:**
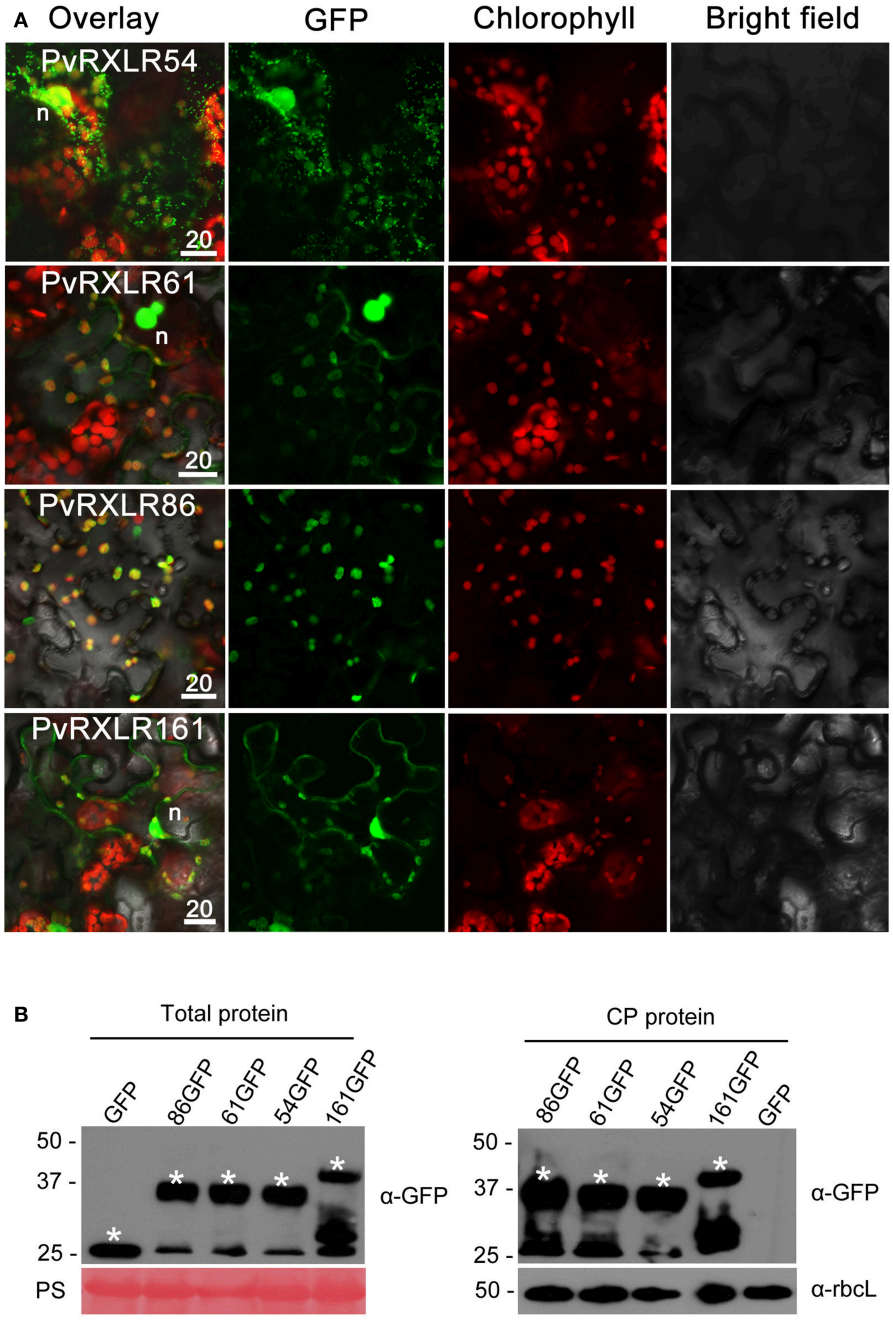
Chloroplastic localization of PvRXLR effectors. **(A)** Effector-GFP fusion constructs were agroinfiltrated into *N. benthamiana* leaves, and the accumulation and distribution pattern of fluorescent protein-tagged effectors has been analyzed by confocal microscopy 36–72 h post-infiltration. Merged images of GFP (green), chlorophyll (red), and bright fields are shown. The yellow fluorescence in the merged image indicates that these effectors are localized in chloroplasts. **(B)** Total leaf protein and chloroplast protein extracts of transformed *N. benthamiana* leaves are separated by using 12% SDS-PAGE, and are transferred onto a nitrocellulose membrane. Immunodetection was performed using an anti-GFP antibody. The ponceau stain and the rubisco (anti-rbcL) are used as loading controls for analyzing the total protein content and chloroplast proteins, respectively. Protein sizes are indicated on the left-hand side of the image in kDa. Asterisks indicate the protein bands discussed in the text. n, nucleus. Scale bar = 20 μm.

Pathogen effectors targeted to host cell organelles are relatively uncommon (Petre et al., 2016). In this study, the following four PvRXLR effectors that were localized to chloroplasts were identified: PvRXLR54 was found to accumulate in chloroplasts, nuclei, and cytosolic punctate structures; PvRXLR61 and PvRXLR161 were both localized in chloroplasts and nuclei; PvRXLR86 was targeted specifically to chloroplasts (Figure 5A). All four of these chloroplastic PvRXLR effectors (54, 61, 161, and 86) are small molecular proteins containing 104, 102, 142, and 198 amino acids, respectively. Amino acid composition analysis revealed that all were rich in leucine (L) and arginine (R), which are known to be important components of chloroplast transit peptides (Bruce, 2000). However, three different software programs (WoLF PSORT, ChloroP, and LOCALIZER) predicted that only PvRXLR86 carried a transit peptide and was potentially targeted to chloroplasts (Table 1). These four effectors showed a very low similarity with each other, and only PvRXLR161 had a predicted RNase H-like domain in the C-terminal region. Protein BLAST results showed that PvRXLR161 was similar to a candidate RXLR effector of *Hyaloperonospora arabidopsis*, but no homologous proteins were identified in the database for the other three PvRXLR effectors.

**Table 1:** Sequence analyses and predicting subcellular localizations of four chloroplast-targeting PvRXLR effectors.

**Effector**	**Amino acid^a^ composition**	**Prediction results**		
		**Wolf PSORTII**	**ChloroP^b^**	**Localizer**
PvRXLR54	A(21.2%), L(15.4%), R(11.5%)	Peroxisome	N	Nucleus
PvRXLR61	L(18.6%), R(13.7%), Q(9.8%)	Nucleus	N	Nucleus
PvRXLR86	S(14.1%), R(10.6%), L(9.6%)	Chloroplast	Chloroplast cTP (18–77)	Chloroplast cTP (35–75)
PvRXLR161	A(10.6%), L(9.9%), S(9.2%)	Chloroplast	N	Nucleus

a *Only the three most abundant amino acids are listed*.

b *N means no chloroplast transit peptide has been predicted*.

To determine if these effectors carried cleavable chloroplast transit peptides and underwent processing *in planta*, we performed anti-GFP western blotting with total proteins and chloroplast proteins extracted from *N. benthamiana* leaves expressing PvRXLR effector-GFP fusion proteins. Figure 5B shows that the positive signals for PvRXLR54-GFP, PvRXLR61-GFP, and PvRXLR161-GFP were in accordance with their predicted molecular weights of 37.5, 37.8, and 41.1 kDa, respectively. However, the positive signal for the PvRXLR86-GFP fusion protein was ~37 kDa, which is significantly smaller than its predicted size of 47.9 kDa. These results suggest that only PvRXLR86 contains an *N*-terminal cleavable transit peptide and can be processed in plant cells, indicating that these effectors utilize different pathways to target chloroplasts.

### PvRXLR54 is targeted to chloroplasts and mitochondria

In order to further identify the cytosolic puncta of PvRXLR54, the Golgi and mitochondria marker proteins GmMan1_1−49_ (the first 49 aa of *Glycine max* α-1,2-mannosidase I) and ScCOX4_1−29_ (the first 29 aa of *Saccharomyces cerevisiae* cytochrome *c* oxidase IV) were selected for labeling these structures, respectively (Köhler et al., 1997; Saint-Jore-Dupas et al., 2006).

**Figure 6:**
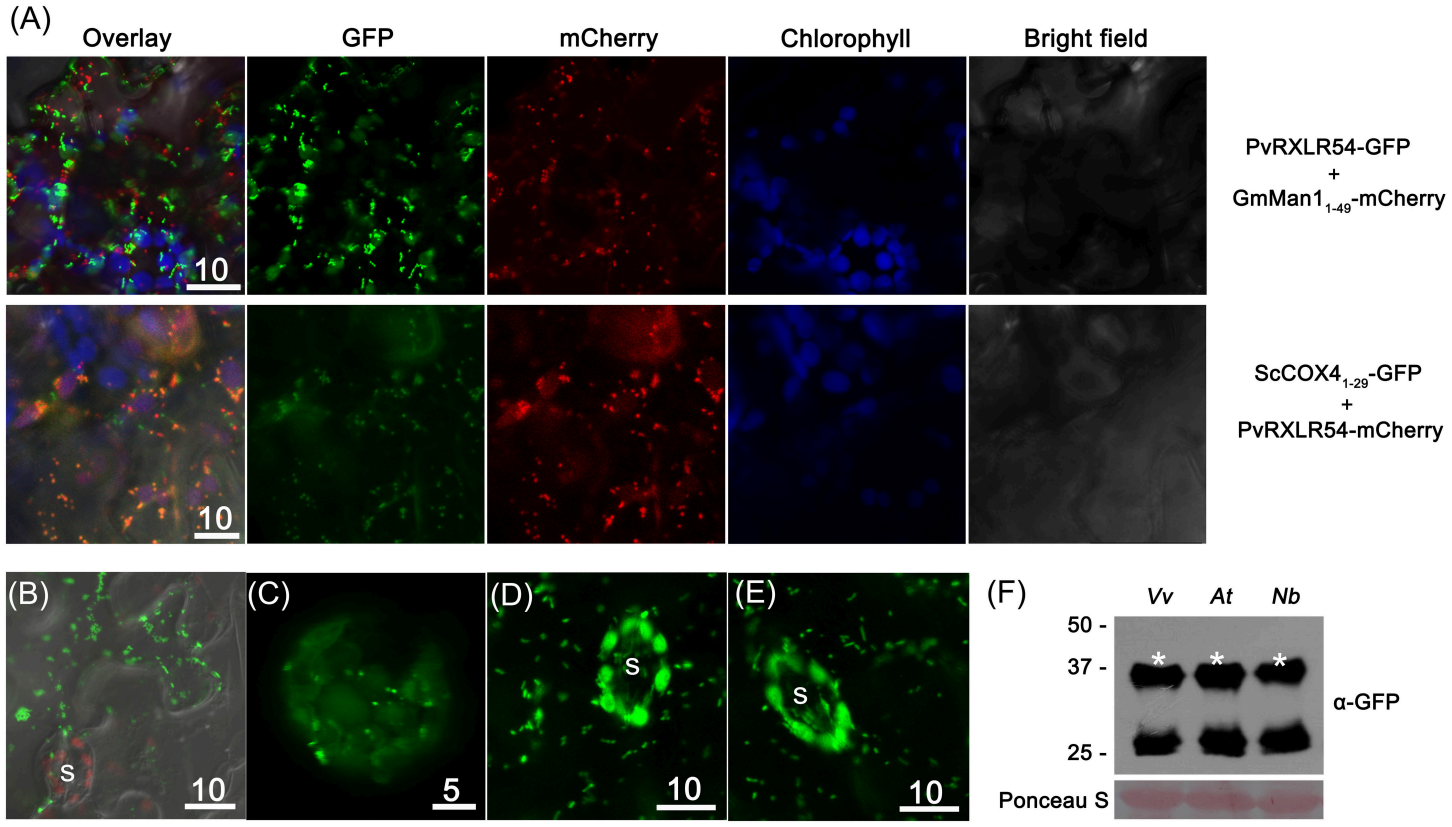
PvRXLR54 is dual-targeted to chloroplasts and mitochondria. Effector-GFP fusion constructs were agroinfiltrated into *N. benthamiana* leaves, and the accumulation and distribution pattern of fluorescent protein-tagged effectors was analyzed by confocal microscopy 36–72 h post-infiltration. **(A)** The mitochondria marker ScCOX4_1−29_-GFP and Golgi marker GmMan1_1−49_-mCherry were transiently co-expressed with PvRXLR54-mCherry and PvRXLR54-GFP, respectively. The yellow fluorescence in the merged image indicates that PvRXLR54 is localized in mitochondria. **(B)** Live-cell imaging of agroinfiltrated *N. benthamiana* leaf epidermal cells. **(C)** Live-cell imaging of *V. vinifera* protoplasts that are transformed with PvRXLR54-GFP. **(D,E)** Live-cell imaging of guard cells of *35S::PvRXLR54-GFP* from transgenic *N. benthamiana* and *A*. *thaliana* T1 seedlings. **(F)** Western blotting of total protein extracted from *V. vinifera* protoplasts and leaves of *A. thaliana* and *N. benthamiana* expressing PvRXLR54-GFP. Protein sizes are indicated on the left-hand side of the image in kDa. Ponceau staining is used to visualize the processes of loading and transfer. Asterisks indicate the protein bands discussed in the text. S, stomata. Scale bar = 5–10 μm.

The co-expression of PvRXLR54_20−104_-GFP and GmMan1_1−49_-mCherry in *N. benthamiana* leaf cells showed that there was no overlap between the fluorescent signals (Figure 6A), indicating that the punctate structures were not Golgi bodies. However, when ScCOX4_1−29_-GFP and PvRXLR54_20−104_-mCherry were co-expressed in *N. benthamiana*, the green and red fluorescence signals overlapped to form an orange-yellow fluorescent signal (Figure 6A), demonstrating that PvRXLR54 was not only targeted to chloroplasts, but was also targeted to mitochondria. Furthermore, mitochondrial targeting of PvRXLR54 was also observed in transformed *N. benthamiana* epidermal cells which lack chloroplasts (Figure 6B). It should be noted that both PvRXLR54_20−104_-GFP and PvRXLR_20−104_-mCherry showed the same subcellular localization, indicating that the fluorescent tags did not influence the subcellular targeting of PvRXLR54.

It has previously been reported that the observed subcellular localization of specific effector proteins might varied, depending on the methods of transformation and the plant species used. For example, AvrRps4-GFP was reported to be localized to the cytoplasm and nucleus in *Agrobacterium*-mediated *N. benthamiana* transient assays, but was found to be localized to chloroplasts in transgenic Arabidopsis (Sohn et al., 2012; Li et al., 2014). To test whether the dual-targeting of PvRXLR54 to the chloroplasts and mitochondria was species-specific, PvRXLR54_20−104_-GFP was transiently expressed in grapevine protoplasts, and stably transformed into *N. benthamiana* and *A. thaliana*. The results showed that the full-length PvRXLR54_20−104_-GFP protein (37.5 kDa) was present in these plant species, and was targeted to both the chloroplast and mitochondria under these conditions (Figures 6C–F). This suggests that the targeting of the PvRXLR54 effector protein to these organelles utilizes a conserved transport pathway in plant cells.

## Discussion

In this study, we have used the heterologous *N. benthamiana* expression system to investigate the biological activity and subcellular localization of candidate RXLR effectors from *P. viticola*. A total of 83 candidate *PvRXLR* genes were successfully cloned from *P. viticola* genomic DNA and functionally characterized. In a previous study, we concluded on the basis of RNA-Seq analysis, that 45 of these *PvRXLR* genes were expressed during infection of grapevine leaves (Yin et al., 2017). We have now confirmed using RT-PCR that a further 33 *PvRXLR* genes are expressed in infected grapevine leaf tissues, indicating that most of the candidate *PvRXLR* genes predicted within the *P. viticola* genome are expressed during infection. The remaining five candidate *PvRXLR* genes are either not expressed or are expressed at levels that could not be detected by RT-PCR. Alternatively, these predicted *PvRXLR* genes might be expressed during other times in the life cycle of this oomycete pathogen, for e.g., during sporulation.

The *N*-terminal secretory signal peptide is a common feature of all effectors, and is generally used as a first pass filter for narrowing down a whole proteome dataset into a shortlist of potential effector candidates. Various techniques have been used to functionally validate the predicted *N*-terminal secretory signal peptides of pathogen effectors including the adenylate cyclase (CyaA) fusion assay, culture filtrate immunoblotting, and live-cell imaging (Sory et al., 1995; Khang et al., 2010; Ribot et al., 2013). However, it is not feasible to use above methods with obligate biotrophs such as *P. viticola*. Therefore, in this study, we utilized the yeast signal sequence trap assay and demonstrated that ~85% of PvRXLR effector signal peptides were able to facilitate the secretion of invertase from yeast cells, confirming that *in silico* prediction tools are capable of predicting the secretome of oomycete biotrophic pathogens such as *P. viticola* with a high degree of accuracy.

The suppression of plant innate immunity is thought to be the primary function of bacterial effectors, and is likely to also be an important activity of effectors secreted by oomycetes, fungi and nematodes. For instance, Fabro et al. (2011) reported that 77% of *H. arabidopsidis* effectors tested could increase *Pseudomonas syringae* pv. *tomato* DC3000-LUX (*Pst-LUX*) growth in Col-0 and suppress *Pst*ΔCEL-induced callose deposition. Germain et al. (2017) concluded that the majority of poplar rust fungus candidate effectors examined were capable of promoting *H. arabidopsidis* growth in Arabidopsis. Our results with PvRXLR effectors indicate that over half of the effectors tested could suppress cell death induced by INF1 and BAX in *N. benthamiana*, which is in line with previous results obtained by Xiang et al. (2016) on a much smaller subset of PvRXLR candidate effectors.

A number of pathogen effectors that cause plant cell death have previously been identified. The best example of plant-specific recognition is the recognition of avirulence effectors either directly or indirectly by nucleotide-binding-leucine-rich-repeat receptors (NLRs), which results in the activation of plant programmed cell death, thus halting the further growth of the pathogens (Whitham et al., 1994; Tang et al., 1996; Dodds et al., 2004; Rehmany et al., 2005; Cesari et al., 2013). Phylogenetic analysis revealed that *Phytophthora* and *Plasmopara* are closely related groups belonging to the order Peronosporales, and share RXLR-type effectors. Thus, some effectors of *Phytophthora* and *Plasmopara* possibly recognize the same protein in *N. benthamiana* and grapevine species. NLR-mediated cell death is generally subtle and rapid; in this study, a very low level of the PvRXLR35 effector could trigger cell death. Hence, we thought that it might be recognized by an unknown NLR that was conserved in *N. benthamiana* and grapevine species. The remaining nine effectors might be recognized by NLRs in *N. benthamiana* as well. Alternatively, one possible explanation is that the high-level expression of these nine PvRXLR proteins in the agroinfiltration assay could lead them to aberrantly bind to proteins, other than their intended targets, leading to unspecific cell death.

Recent studies have demonstrated that many oomycete effectors are targeted to the plant nucleus. For example, 66% of HaRxLR effectors examined were targeted to either the nucleus specifically or to the nucleus and cytoplasm (Caillaud et al., 2012). Schornack et al. (2010) also showed that a subset of Crinkler (CRN) effectors from *P. infestans* were targeted to the nucleus. Our findings are in line with these observations, as over half of the PvRXLR effectors investigated were targeted to the nucleus. In addition, we observed that multiple PvRXLR effectors showed sub-nuclear localizations with different features. Although the mechanism of protein sub-nuclear distribution remains unknown, it has been reported that many pre-mRNA splicing factors, kinases, and phosphatases could localize to nuclear speckles, and were involved in regulating transcription and pre-mRNA processing (Lamond and Spector, 2003; Spector and Lamond, 2011). Thus, we speculate that these PvRXLR effectors might be involved in the regulation of gene expression in the host.

To date, no oomycete effectors have been confirmed to target chloroplasts or mitochondria, despite the fact that several large-scale subcellular localization screening experiments have been conducted with a range of oomycete pathogens. In this study, we identified four PvRXLR effectors that were localized to chloroplasts, including PvRXLR54 dual-targeted to chloroplasts and mitochondria. One explanation for this might be that previous studies used *N*-terminally tagged GFP-effector fusions, which might have impaired the proper translocation of these effector proteins into the organelles, whereas our experiments used C-terminal effector-GFP fusions. It is unclear how these PvRXLR effectors are transported into host organelles. Most plant chloroplast proteins utilize classical cleavable transit peptides to facilitate import into organelles, while some proteins lacking cleavable transit peptides might target the chloroplasts through the endomembrane system (Chen et al., 2004; Kleffmann et al., 2004; Villarejo et al., 2005; Nanjo et al., 2006).

It will be interesting to examine how the recognition and uptake of PvRXLR54 by the transport systems of both chloroplasts and mitochondria are possible. One explanation is that the effector contains a multipartite transit peptide, in which different segments are recognized by different receptors in each of the mitochondria and plastid import machineries (Carrie and Small, 2013; Baudisch et al., 2014). For example, alpha-MPP2 (one of the two isoforms of the substrate-binding subunit of mitochondria-processing peptidase in Arabidopsis) is composed of three functionally separated domains and carries a dual-target signal. The 29 *N*-terminal residues of alpha-MPP2 mediate mitochondrial targeting, whereas chloroplast transport requires the entire *N*-terminal region (Baudisch and Klösgen, 2012). Further studies are necessary to identify the different motifs within PvRXLR54 that control the targeting of this effector to different subcellular organelles.

In conclusion, this study has increased our understanding of the molecular mechanisms of pathogenicity of *P. viticola*. The identification of PvRXLR effectors targeted to chloroplasts and mitochondria have also expanded our understanding of the biological activity of oomycete effectors. Future studies will focus on the identification of the host targets of these PvRXLR effectors, to understand how these secreted pathogenic proteins are able to modulate the innate immune responses of the plant.

## Author contributions

All authors contributed to this work. JL conceived and supervised the research, and SS assisted the project operation. XL amplified effector genes, constructed the vectors, transformed *Agrobacterium*, and performed yeast signal peptide trap assay. JX cultivated *N. benthamiana* seedlings. XL and YL generated stable transgenic plants and performed agroinfiltration, protoplasts expression experiments, confocal microscopy, western blotting, and data analysis. ID, LY, and JQ performed bioinformatics analysis. YL, XL, and JL wrote the manuscript. JL, ID, and SS edited the manuscript.

### Conflict of interest statement

The authors declare that the research was conducted in the absence of any commercial or financial relationships that could be construed as a potential conflict of interest.

## Supplementary material

Figure S1 RT-PCR expression analysis of candidate *PvRXLR* genes. Fifty nanograms of RNA were isolated from infected leaves and transcribed to cDNA after DNase treatment. Full-length primers were used to amplify effector genes from cDNA; the corresponding RT-PCR results are shown. White asterisks indicate that the bands are specific for effectors, while red asterisks indicate non-specific bands. The housekeeping gene *Vvactin* was used as a control for cDNA synthesis. M, DNA marker.

Figure S2 Functional validation of predicted signal peptides of PvRXLR effectors in yeast. The predicted effector signal peptide sequences were fused in frame to the invertase gene in the pSUC2 vector, and transformed into yeast YTK12 (invertase negative). Yeast is able to grow on YPRAA only if the invertase protein is secreted. CK–, untransformed YTK12. CK+, Avr1b.

Figures S3–S5 Phenotypes observed upon expression of PvRXLR effectors in *N. benthamiana* leaves. Effector and GFP control constructs were agroinfiltrated into the left and right hand panels of *N. benthamiana* leaves, respectively. One day later, the infiltration sites were re-infiltrated with INF1. Leaves were photographed and analyzed 4–6 days after infiltration. Similar results were obtained from at least two independent experiments. **(S3)** Symptoms observed with PvRXLR effectors that suppress INF-induced cell death. **(S4A)** Symptoms observed with PvRXLR effectors that partially suppress INF-induced cell death. **(S4B)** Symptoms observed with PvRXLR effectors that failed to suppress INF-induced cell death. **(S4C)** Symptoms observed with PvRXLR effectors that enhanced cell death. **(S5)** A graphical summary of the symptoms observed in (**S3,S4)**.

Figures S6–S10 Subcellular localization of PvRXLR effectors in *N. benthamiana* leaf cells. Effector-GFP fusion constructs were agroinfiltrated into *N. benthamiana* leaves, and the accumulation and distribution pattern of fluorescent protein-tagged effectors was analyzed by confocal microscopy 36–72 h post-infiltration. **(S6)** A graphical summary of the observed subcellular localization of PvRXLR effector-GFP fusion proteins. **(S7)** PvRXLR effectors targeted to the nucleus and cytoplasm. **(S8)** PvRXLR effectors targeted solely to the nucleus. **(S9)** PvRXLR effectors targeted to diverse sub-nuclear localizations. **(A)**, Nucleolus; **(B)**, Irregular structures within the nucleus. **(S10)** PvRXLR effectors targeted to plant cell membranes. Scale bar = 5–20 μm.

Figure S11 Live-cell imaging of effector-GFP fusions in transgenic *A. thaliana* seedlings. Confocal images of leaf epidermal cells of 10 days old transgenic plantlets expressing candidate effectors fused to GFP in a Col-0 genetic background. PvRXLR47, 126, and 151 are labeled to the endoplasmic reticulum (ER) (around the nucleus); PvRXLR85, 143, and 154 are labeled to the plasma membrane; the remaining effectors showed nucleocytoplasmic localizations. Scale bar = 10–20 μm.

Table S1 Amino acid sequences of the selected 83 effectors.

Table S2 List of primers used in this study.
