# Supplementary figures and images for: In Planta Functional Analysis and Subcellular Localization of the Oomycete Pathogen Plasmopara viticola Candidate RXLR Effector Repertoire
Source: Front Plant Sci. 2018 Apr 13;9:286. doi: 10.3389/fpls.2018.00286 (PMC5908963; doi:10.3389/fpls.2018.00286)

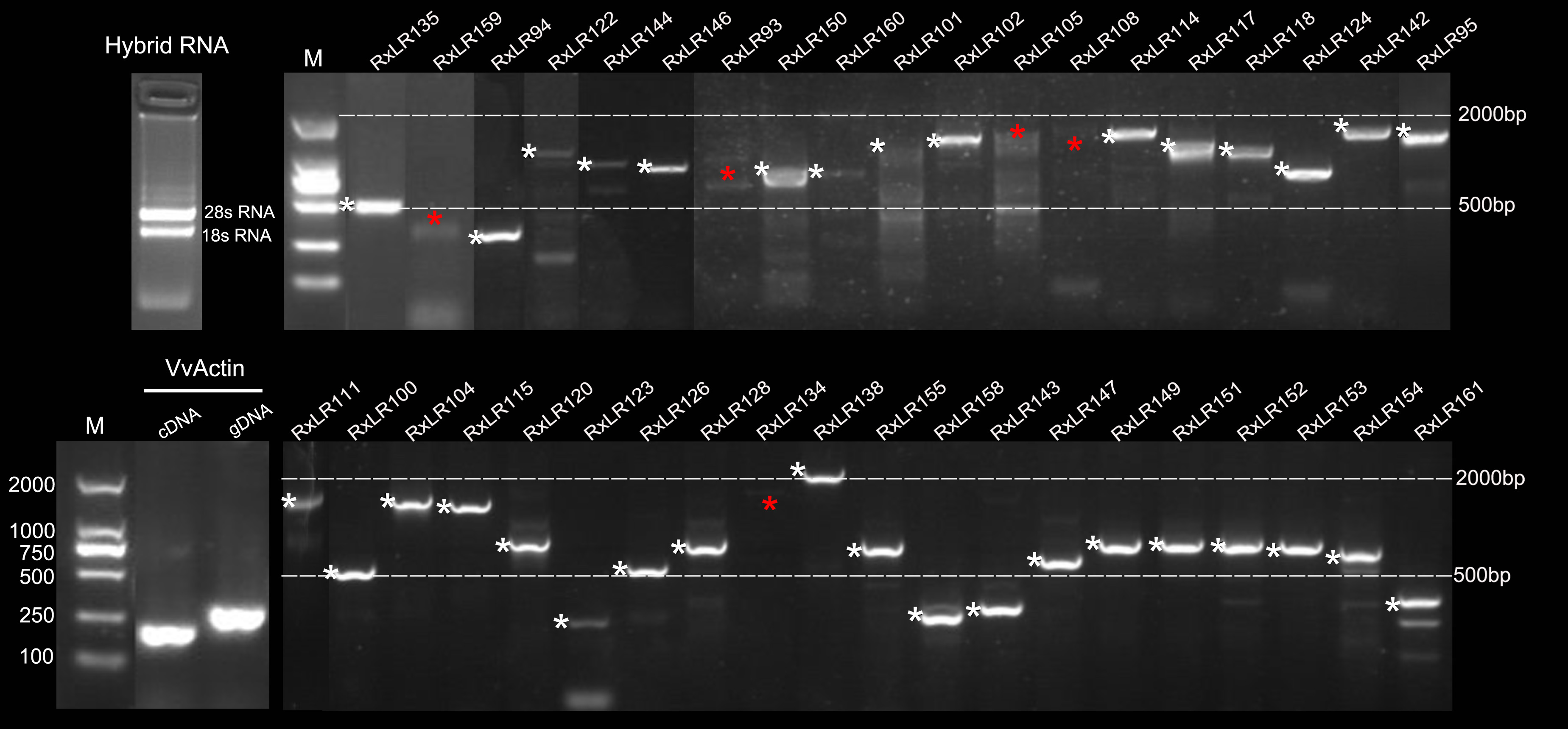

Supplement: Figure S1 — RT-PCR expression analysis of candidate PvRXLR genes. Fifty nanograms of RNA were isolated from infected leaves and transcribed to cDNA after DNase treatment. Full-length primers were used to amplify effector genes from cDNA; the corresponding RT-PCR results are shown. White asterisks indicate that the bands are specific for effectors, while red asterisks indicate non-specific bands. The housekeeping gene Vvactin was used as a control for cDNA synthesis. M, DNA marker. [file Image1.JPEG]

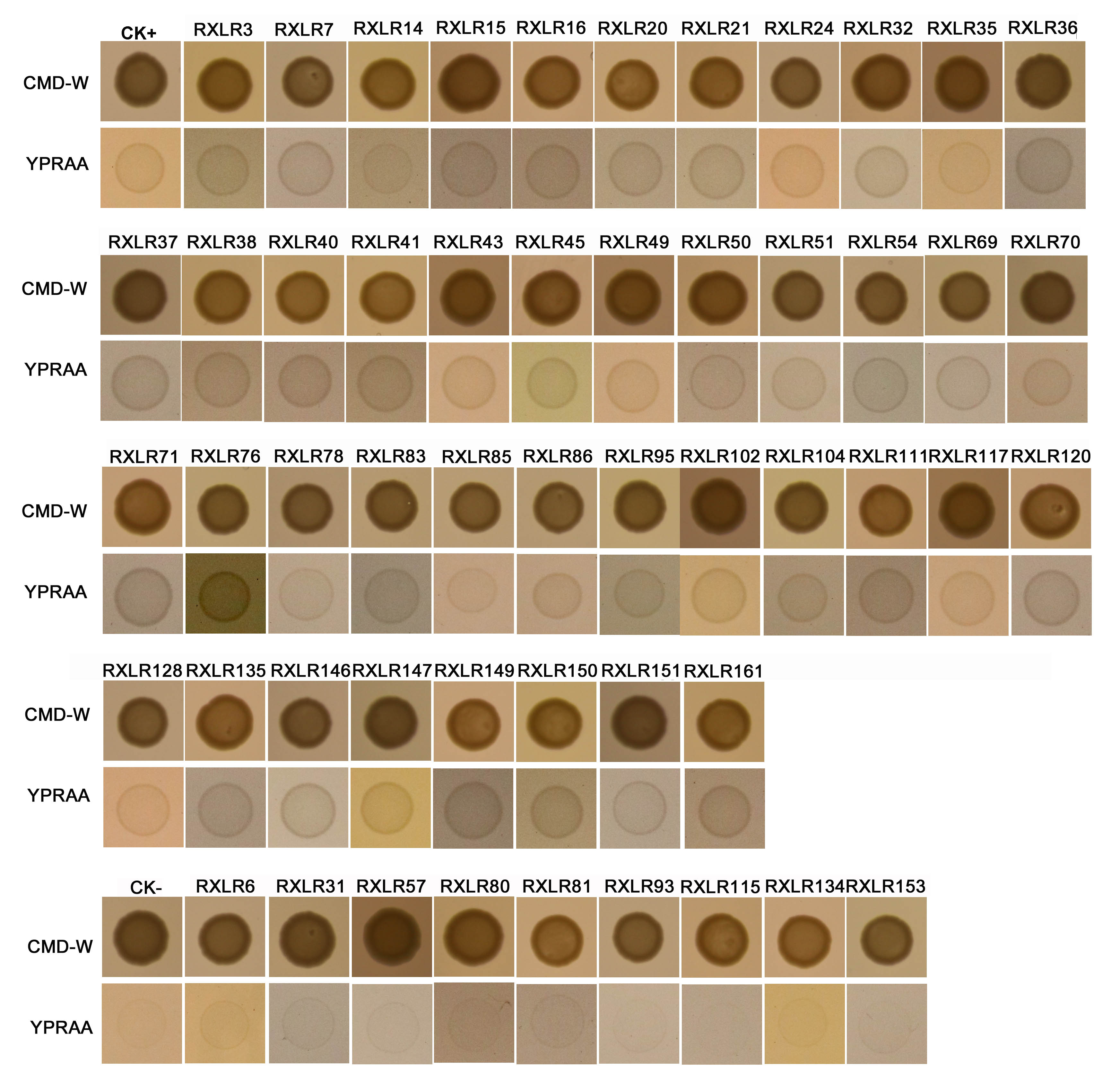

Supplement: Figure S2 — Functional validation of predicted signal peptides of PvRXLR effectors in yeast. The predicted effector signal peptide sequences were fused in frame to the invertase gene in the pSUC2 vector, and transformed into yeast YTK12 (invertase negative). Yeast is able to grow on YPRAA only if the invertase protein is secreted. CK–, untransformed YTK12. CK+, Avr1b. [file Image2.JPEG]

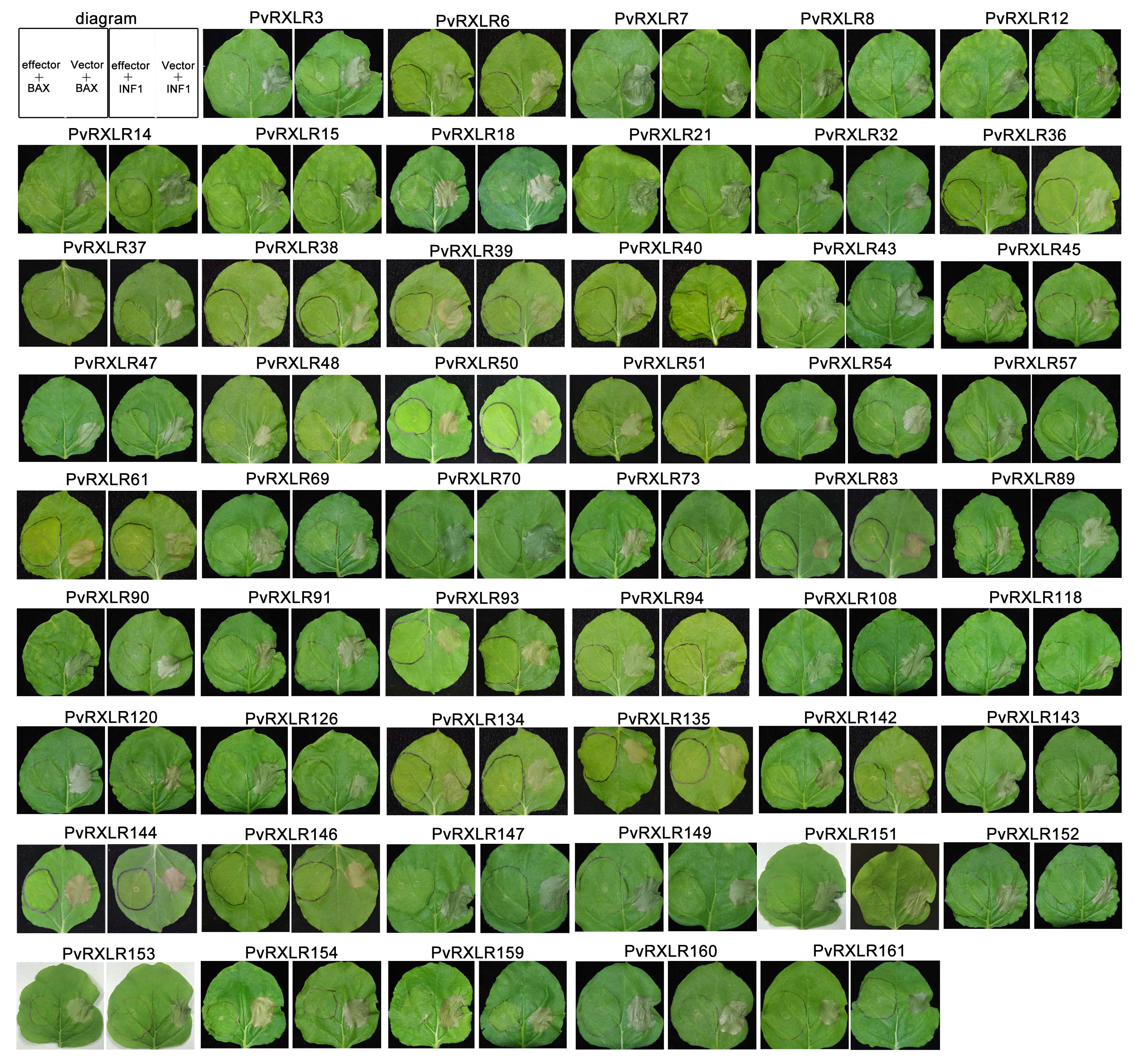

Supplement: Figures S3–S5 — Phenotypes observed upon expression of PvRXLR effectors in N. benthamiana leaves. Effector and GFP control constructs were agroinfiltrated into the left and right hand panels of N. benthamiana leaves, respectively. One day later, the infiltration sites were re-infiltrated with INF1. Leaves were photographed and analyzed 4–6 days after infiltration. Similar results were obtained from at least two independent experiments. (S3) Symptoms observed with PvRXLR effectors that suppress INF-induced cell death. (S4A) Symptoms observed with PvRXLR effectors that partially suppress INF-induced cell death. (S4B) Symptoms observed with PvRXLR effectors that failed to suppress INF-induced cell death. (S4C) Symptoms observed with PvRXLR effectors that enhanced cell death. (S5) A graphical summary of the symptoms observed in (S3,S4). [file Image3.JPEG]

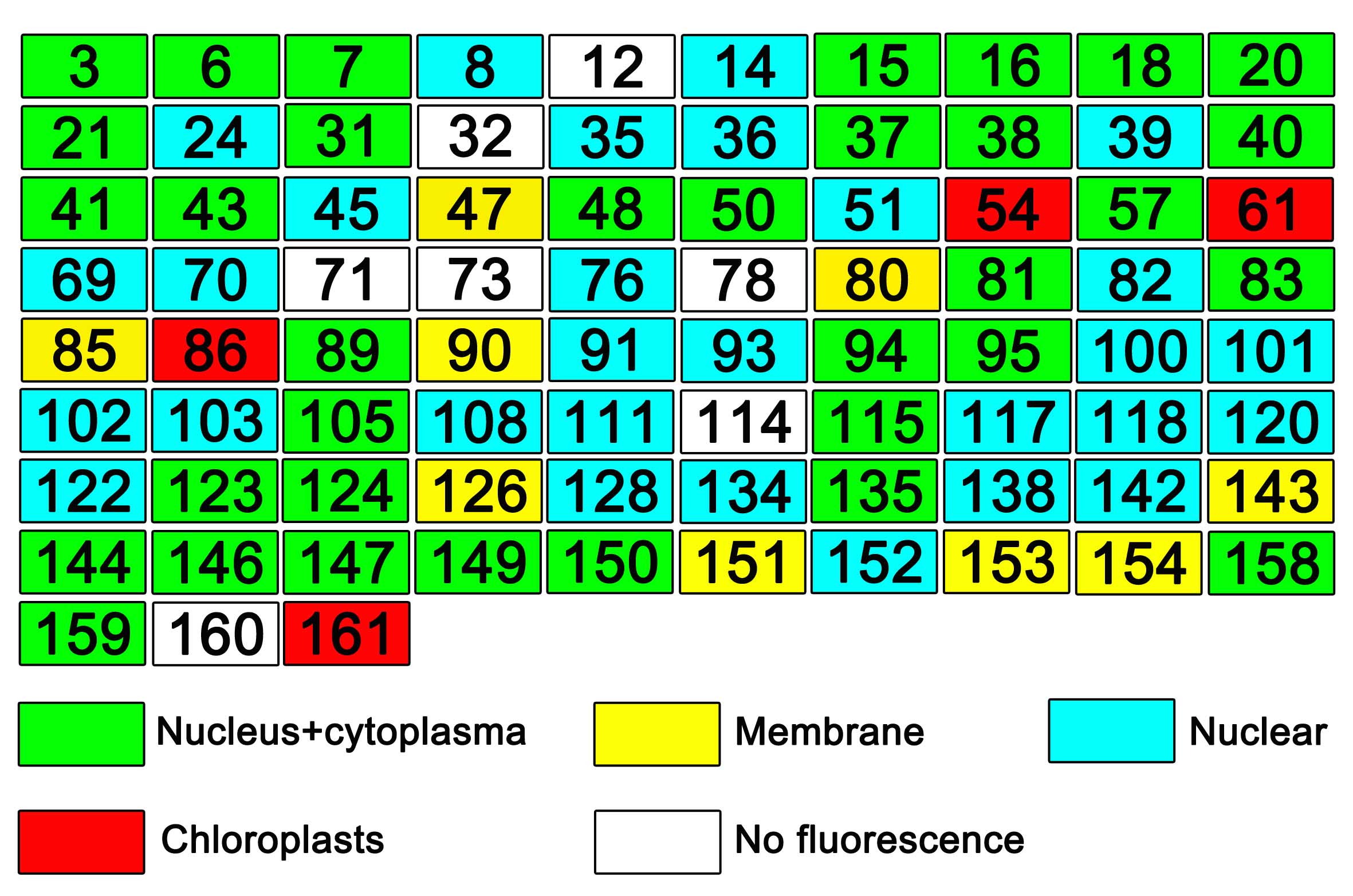

Supplement: Figures S6–S10 — Subcellular localization of PvRXLR effectors in N. benthamiana leaf cells. Effector-GFP fusion constructs were agroinfiltrated into N. benthamiana leaves, and the accumulation and distribution pattern of fluorescent protein-tagged effectors was analyzed by confocal microscopy 36–72 h post-infiltration. (S6) A graphical summary of the observed subcellular localization of PvRXLR effector-GFP fusion proteins. (S7) PvRXLR effectors targeted to the nucleus and cytoplasm. (S8) PvRXLR effectors targeted solely to the nucleus. (S9) PvRXLR effectors targeted to diverse sub-nuclear localizations. (A), Nucleolus; (B), Irregular structures within the nucleus. (S10) PvRXLR effectors targeted to plant cell membranes. Scale bar = 5–20 μm. [file Image6.JPEG]

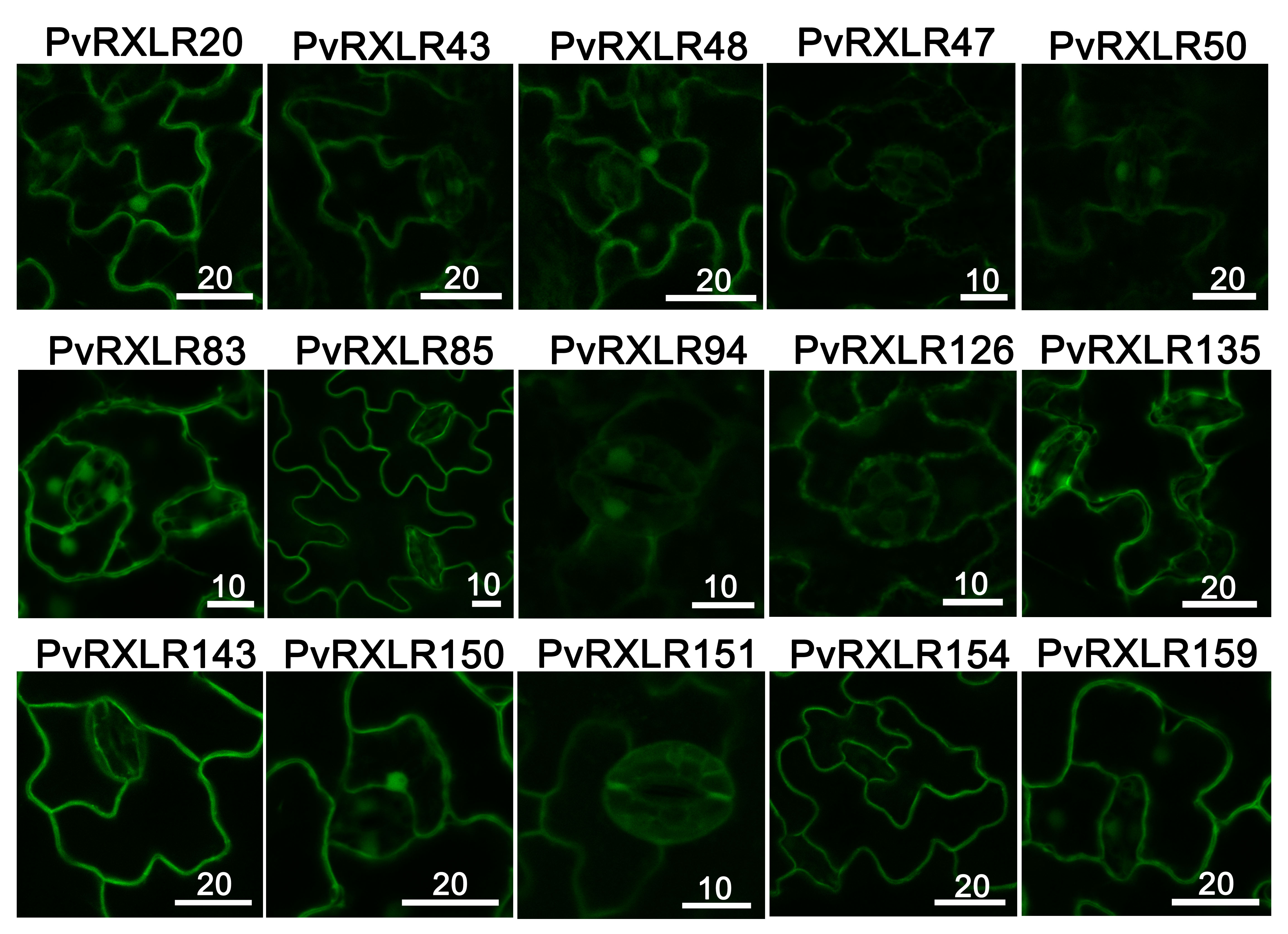

Supplement: Figure S11 — Live-cell imaging of effector-GFP fusions in transgenic A. thaliana seedlings. Confocal images of leaf epidermal cells of 10 days old transgenic plantlets expressing candidate effectors fused to GFP in a Col-0 genetic background. PvRXLR47, 126, and 151 are labeled to the endoplasmic reticulum (ER) (around the nucleus); PvRXLR85, 143, and 154 are labeled to the plasma membrane; the remaining effectors showed nucleocytoplasmic localizations. Scale bar = 10–20 μm. [file Image11.JPEG]

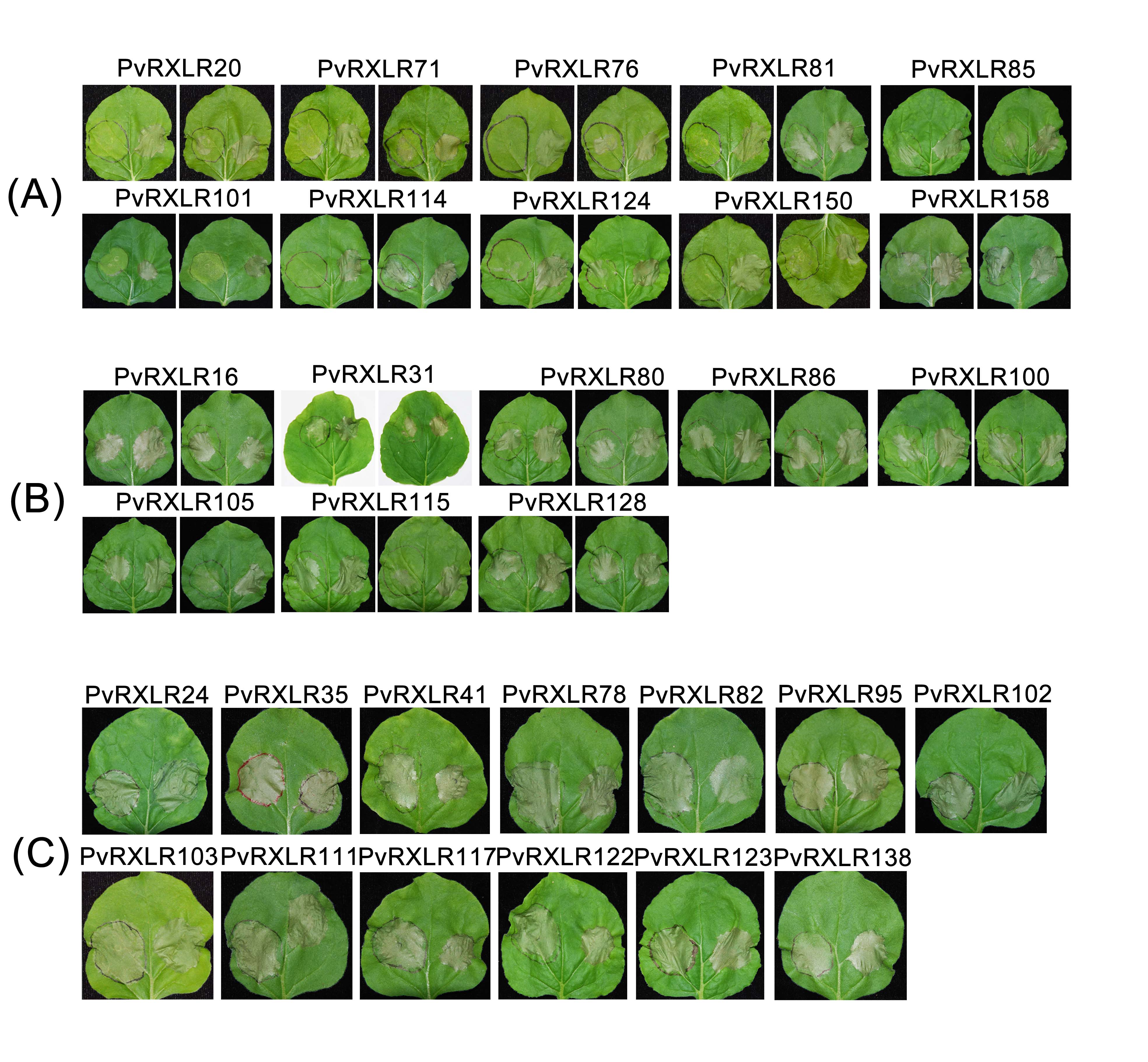

Supplement: Supplementary file 8 [file Image4.JPEG]

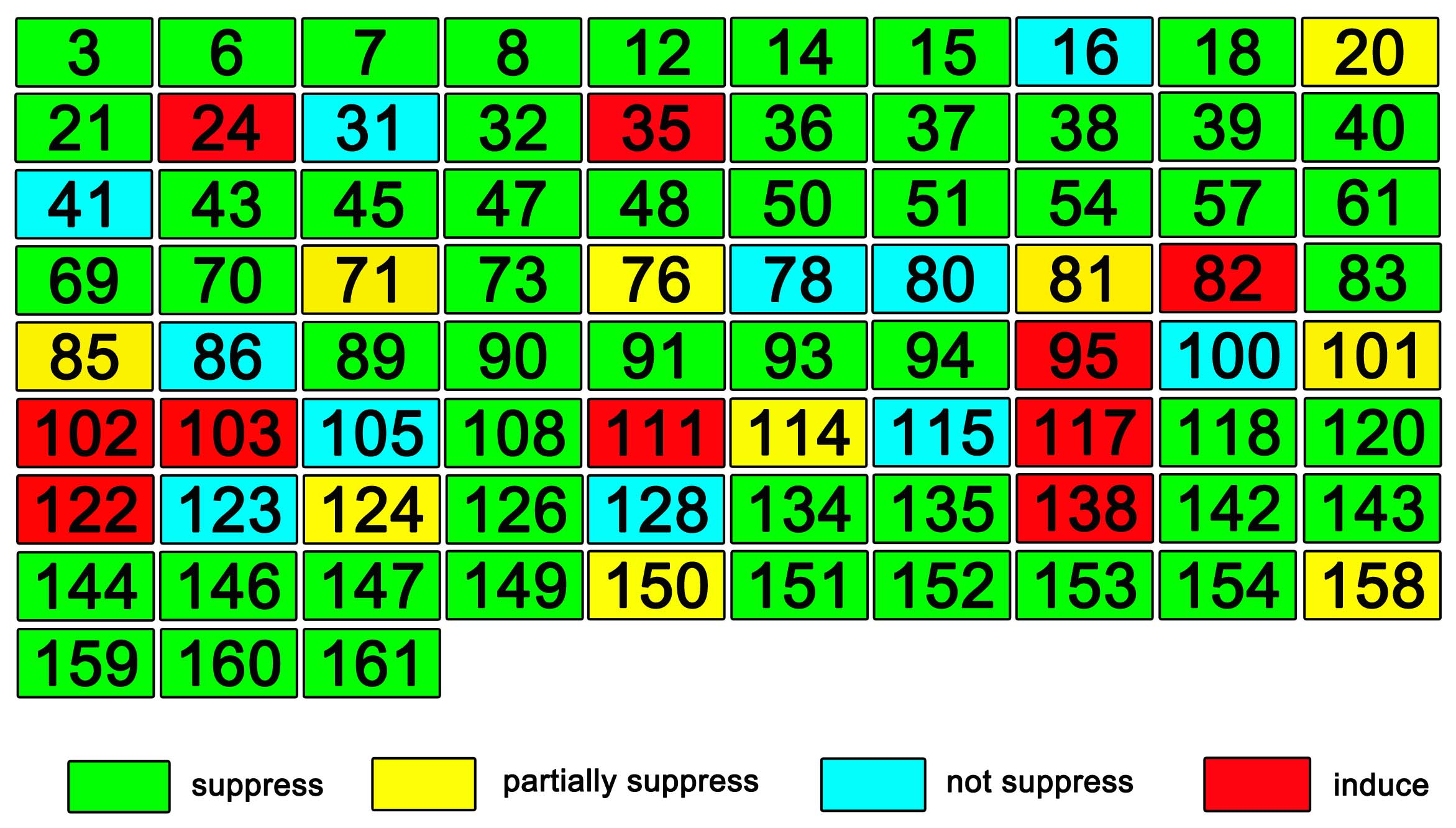

Supplement: Supplementary file 9 [file Image5.JPEG]

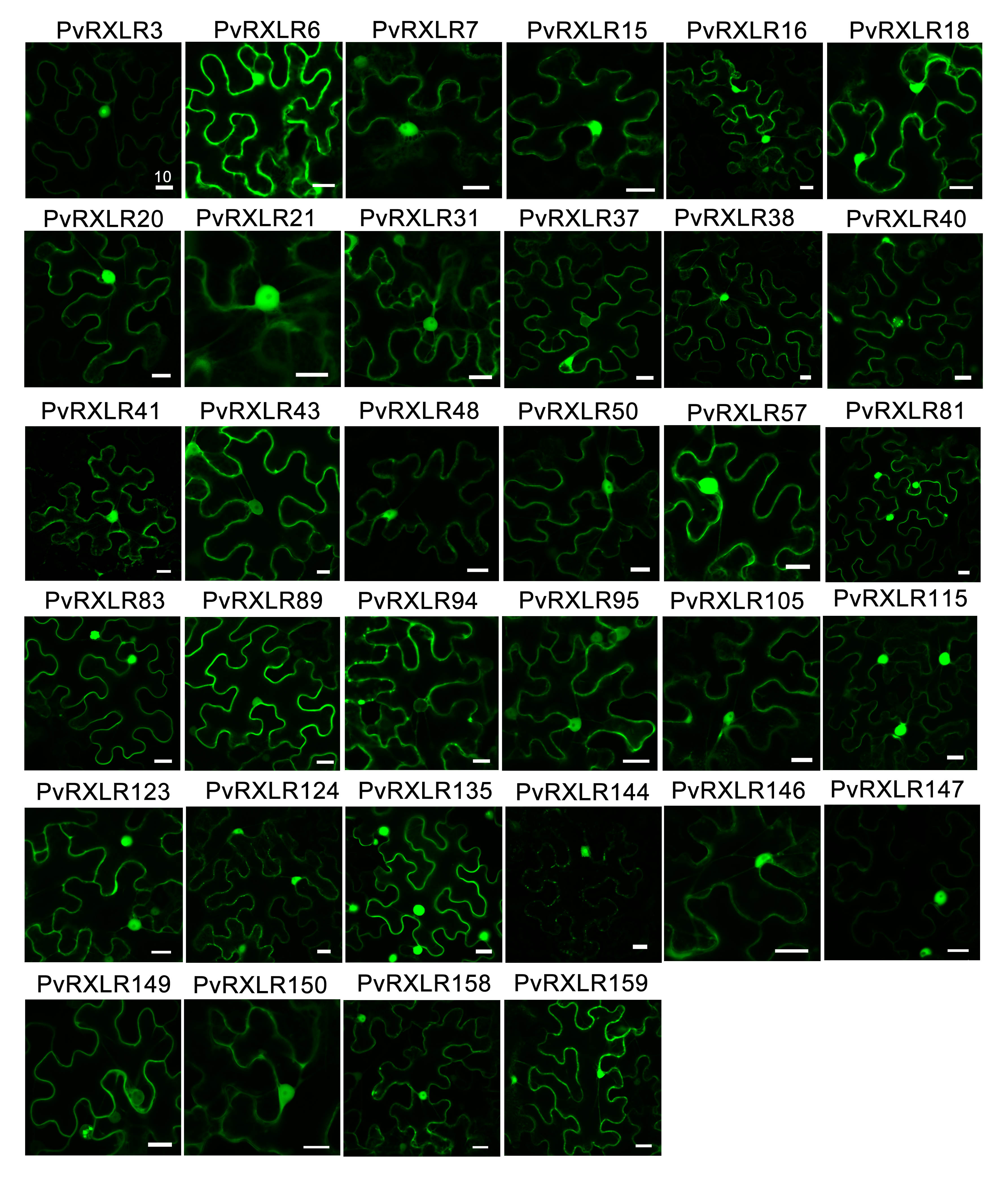

Supplement: Supplementary file 10 [file Image7.JPEG]

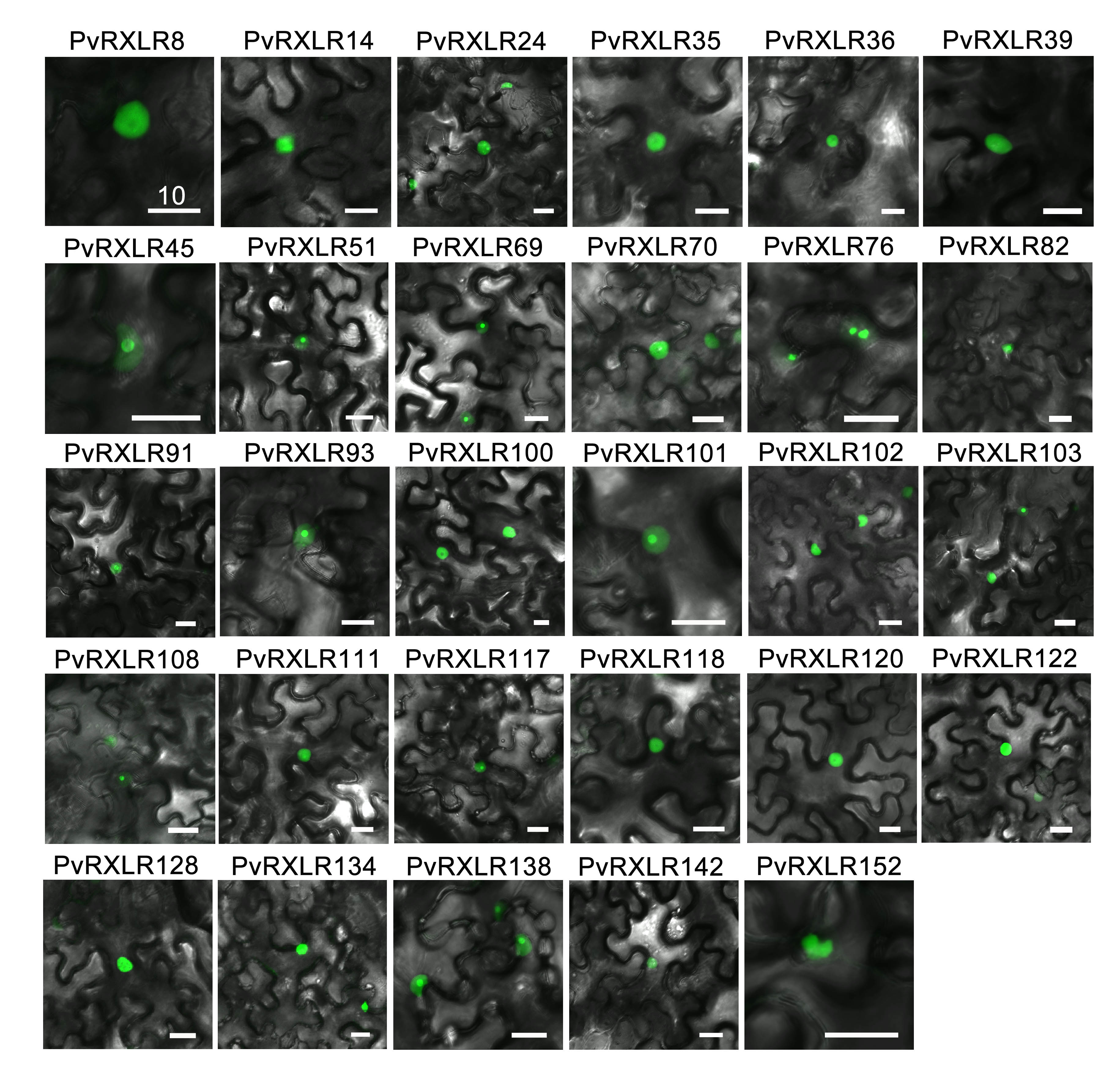

Supplement: Supplementary file 11 [file Image8.JPEG]

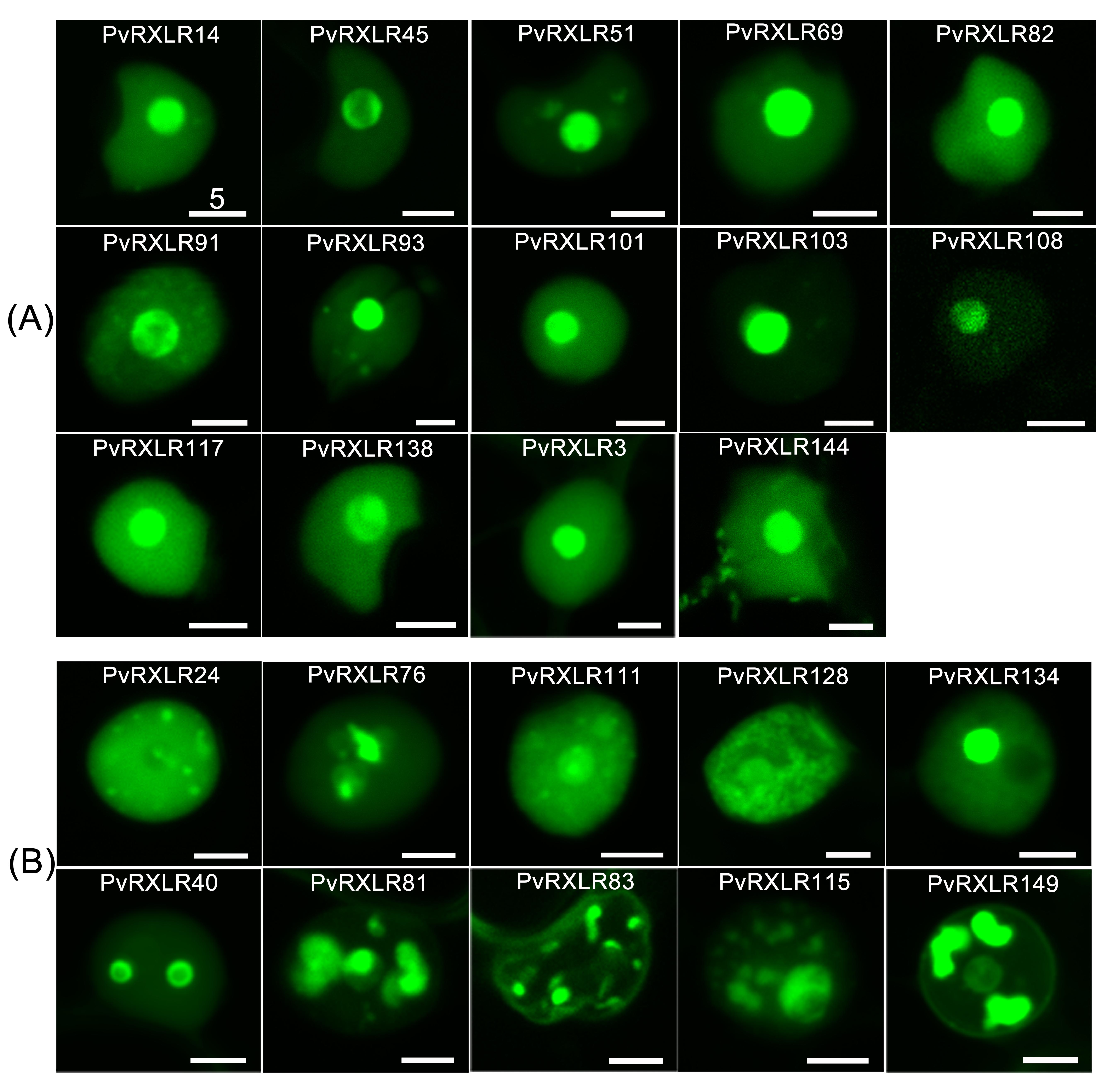

Supplement: Supplementary file 12 [file Image9.JPEG]

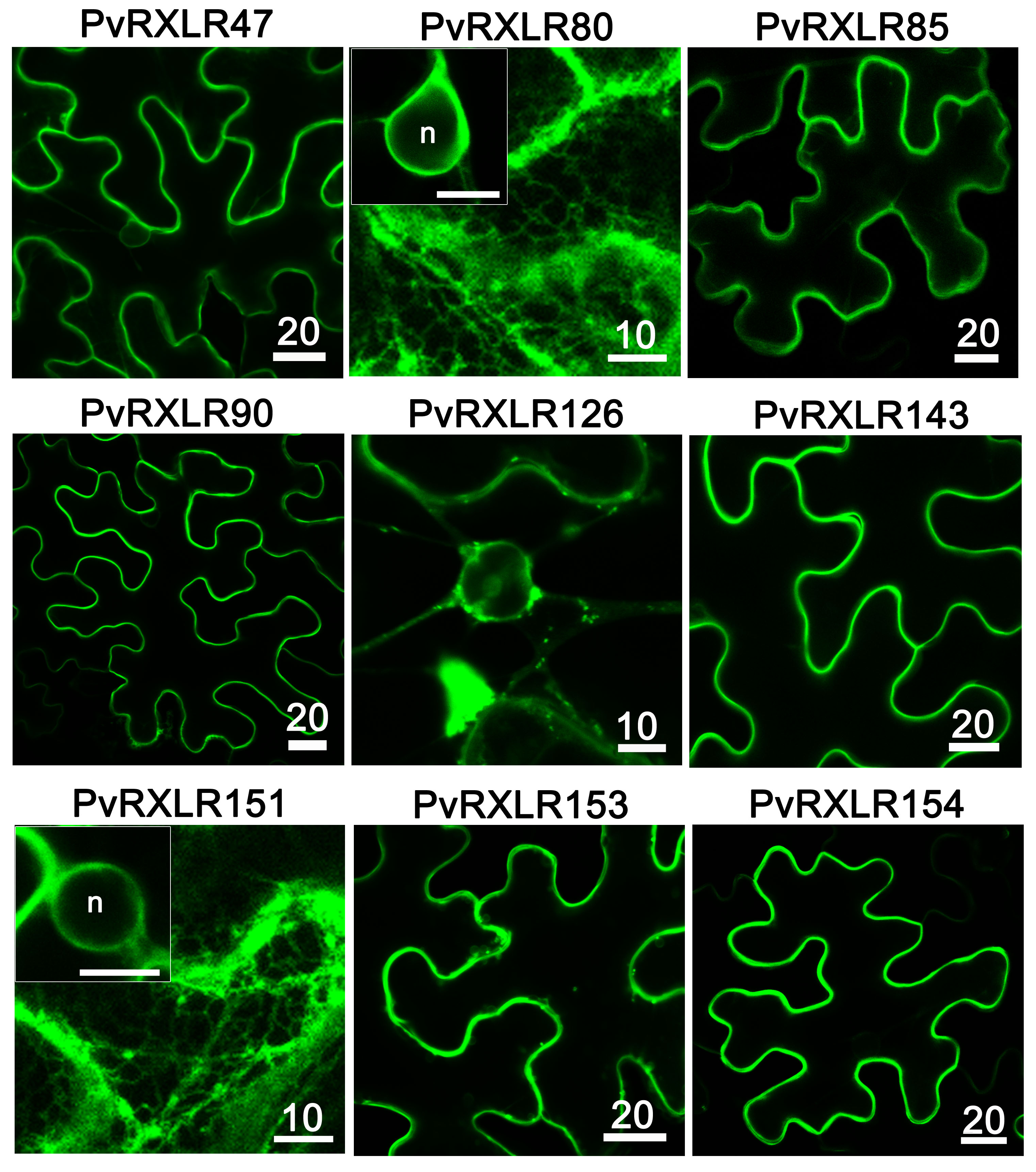

Supplement: Supplementary file 13 [file Image10.JPEG]
